# eIF3k Domain-Containing Protein Regulates Conidiogenesis, Appressorium Turgor, Virulence, Stress Tolerance, and Physiological and Pathogenic Development of *Magnaporthe oryzae Oryzae*

**DOI:** 10.3389/fpls.2021.748120

**Published:** 2021-10-18

**Authors:** Lili Lin, Jiaying Cao, Anqiang Du, Qiuli An, Xiaomin Chen, Shuangshuang Yuan, Wajjiha Batool, Ammarah Shabbir, Dongmei Zhang, Zonghua Wang, Justice Norvienyeku

**Affiliations:** ^1^State Key Laboratory of Ecological Pest Control for Fujian and Taiwan Crops, Fujian Agriculture and Forestry University, Fuzhou, China; ^2^Fujian University Key Laboratory for Plant-Microbe Interaction, Fujian Agriculture and Forestry University, Fuzhou, China; ^3^Key Laboratory of Green Prevention and Control of Tropical Plant Diseases and Pests, Ministry of Education, College of Plant Protection, Hainan University, Haikou, China; ^4^Institute of Oceanography, Minjiang University, Fuzhou, China

**Keywords:** *Magnaporthe oryzae Oryzae*, ribosomal RNA (rRNA), non-essential eIF3 complex, appressorium, nutrition starvation

## Abstract

The eukaryotic translation initiation factor 3 (eIF3) complex consists of essential and non-essential sub-complexes. Non-essential eIF3 complex subunits, such as eIF3e, eIF3j, eIF3k, and eIF3l, modulate stress tolerance and enhance the lifespan of *Neurospora crassa* and *Caenorhabditis elegans*. However, there is limited knowledge of the role of the non-essential eIF3 sub-complex in the pathophysiological development of plant fungal pathogens. Here, we deployed genetic and biochemical techniques to explore the influence of a hypothetical protein containing eIF3k domain in *Magnaporthe oryzae Oryzae* (*MoOeIF3k*) on reproduction, hyphae morphogenesis, stress tolerance, and pathogenesis. Also, the targeted disruption of *MoOeIF3k* suppressed vegetative growth and asexual sporulation in Δ*Mo*O*eif3k* strains significantly. We demonstrated that *MoOeIF3k* promotes the initiation and development of the rice blast disease by positively regulating the mobilization and degradation of glycogen, appressorium integrity, host penetration, and colonization during host–pathogen interaction. For the first time, we demonstrated that the eIF3k subunit supports the survival of the blast fungus by suppressing vegetative growth and possibly regulating the conversions and utilization of stored cellular energy reserves under starvation conditions. We also observed that the deletion of *MoOeIF3k* accelerated ribosomal RNA (rRNA) generation in the Δ*Mo*O*eif3k* strains with a corresponding increase in total protein output. In summary, this study unravels the pathophysiological significance of eIF3k filamentous fungi. The findings also underscored the need to systematically evaluate the individual subunits of the non-essential eIF3 sub-complex during host–pathogen interaction. Further studies are required to unravel the influence of synergetic coordination between translation and transcriptional regulatory machinery on the pathogenesis of filamentous fungi pathogens.

## Introduction

Diverse regulatory mechanisms, such as transcription, splicing, messenger RNA (mRNA) structure, mRNA stability, translation, and protein degradation, crucially regulate gene expression in eukaryotic organisms (Arraiano and Maquat, 2003; Unbehaun et al., 2004; Boo and Kim, 2020). Eukaryotic translation initiation factors (eIFs) play a fundamental and indispensable role in regulating gene expression in eukaryotic cells at the translational level by mediating the onset of mRNA translation into proteins (Masutani et al., 2007; Lee et al., 2015; Smith et al., 2016).

The eukaryotic translation initiation factor 3 (eIF3) complex is currently the largest translational initiation complex identified in eukaryotes. To initiate the process of translation, eIF3 binds to 40S and, in turn, stimulates the recruitment and binding of other initiation factors, such as the eIF2-GTP-Met-tRNAiMet ternary complex (TC) to form the 43S pre-initiation complex (Phan et al., 2001). Once bound to sites proximal to the “E” (deacylated transfer RNA, tRNA) site on the small ribosomal subunit (40S), eIF3 prevents the premature attachment of large ribosomal subunits (60S) to the 43S complex prior to the binding of mRNA to the P (peptidyl) site on the 40S of the 43S complex. Also, the interaction of eIf3 with the eIF2/GTP/Met-tRNAi TC promotes ribosomal recycling by inhibiting the interaction between the 60S subunits and the 43S complex after recycling (Jackson et al., 2010; Hashem et al., 2013). The regulatory influence of the eIF3 complex extends across multiple stages of the initiation process, such as charging of tRNAs associated with the 40S ribosomal subunit and also facilitates the loading of charged 40S onto the mRNAs harboring the methylated guanosine cap at the 5′ untranslated region (UTR) by forming a complex with eIF4F (Jeong et al., 2019; Wolf et al., 2020). The eIF3 complex also influences both the scanning and the recognition of the start codon on the mRNA (Unbehaun et al., 2004; Jackson et al., 2010).

Furthermore, eIF3 promotes ribosomal recycling by mediating the post-termination dissociation of 60S subunits from the 40S subunit (Siridechadilok et al., 2005). Meanwhile, studies have shown that mRNAs from medically important viruses, such as the hepatitis C virus (HCV) and classical swine fever virus (CSFV), possess internal ribosome entry sites (IRESs) that interact with subunits of the eIF3 complex. Interaction between IRES-eIF3 in the mRNA 5′-cap region and the cap-binding complex results in the initiation of the translation process (Siridechadilok et al., 2005; Valášek et al., 2017).

Currently, eIF3 subunits are classified as essential (conserved, thus, present in all eukaryotes) and non-essential (non-conserved across eukaryotes) (Smith et al., 2016; Wolf et al., 2020). In mammals, the eIF3 complex consists of 11–13 subunits (eIF3a, eIF3b, eIF3c, eIF3d, eIF3e, eIF3f, eIF3g, eIF3h, eIF3i, eIF3j, eIF3k, eIF3n, and eIF3m). The eIF3 complex in *Saccharomyces cerevisiae*, however, comprises five subunits (eIF3a, eIF3b, eIF3c, eIF3g, and eIF3i), which, together with other conserved eIF3 subunits, such as eIF3d, eIF3m, and eIF3n, constitute the essential/core eIF3 complex subunits (eIF3a, eIF3b, eIF3c, eIF3d, eIF3g, eIF3i, eIF3m, and eIF3n), and the remaining five (eIF3e, eIF3h, eIF3j, eIF3k, and eIF3l) form the non-essential eIF3 complex (Dong and Zhang, 2006; Valášek, 2012; Marchione et al., 2013). Studies have shown that eight subunits (eIF3a, eIF3c, eIF3e, eIF3f, eIF3h, eIF3k, eIF3l, and eIF3m) of the eIF3 complex form an octamer. While the three other subunits (eIF3b, eIF3g, and eIF3i) consititute the yeast-like core (YLC) in association with the C-terminal region eIF3a (Valášek et al., 2017), the regulation of protein biosynthesis at the translational level through the deregulation of eIF3 has been shown to impact negatively on both physiological and pathological development of eukaryotes (Marchione et al., 2013).

The individual subunits of the eIF3 complex (eIF3a, eIF3b, eIF3c, eIF3e, eIF3h, and eIF3i) assume an additional role in regulating the translational initiation of specific mRNAs encoding proteins that promote cell growth and hence interfere in the development of cancer and tumor cells in humans (Dong and Zhang, 2006).

Previous revelations that the targeted disruption of eIF3k and eIF3l subunits of the eIF3 complex in *Caenorhabditis elegans* increased the lifespan and rendered the defective strains immune to endoplasmic reticulum (ER) stress further support our earlier position that some subunits of the eIF3 complex play a dispensable role in the initiation of the translation process or assume roles that are not directly related to the initiation of translation (Sha et al., 2009).

Ribonucleic acid-dependent processes, such as eIF3-mediated regulation and translational initiation of mRNAs, significantly influence both the vegetative growth and pathogenesis of fungi pathogens (Alves, 2014; Gunišová et al., 2018). However, there is limited evidence on direct or indirect contributions of individual subunits of the non-conserved eIF3 complex to the physiological and pathological development of the rice blast fungus (*Magnaporthe oryzae Oryzae*). Here, we functionally characterized the putative eIF3 subunit k (*MoOeIF3k*) in the physiological and pathogenesis of the economically destructive rice blast pathogen using molecular genetic techniques. These investigations showed that eIF3k crucially modulates the pathogenic development and survival of the rice blast fungus under starvation conditions.

## Results

### Identification, Phylogeny Analysis, and Targeted Gene Replacement of *Mo*O*eIF3K*

**FIGURE 1:**
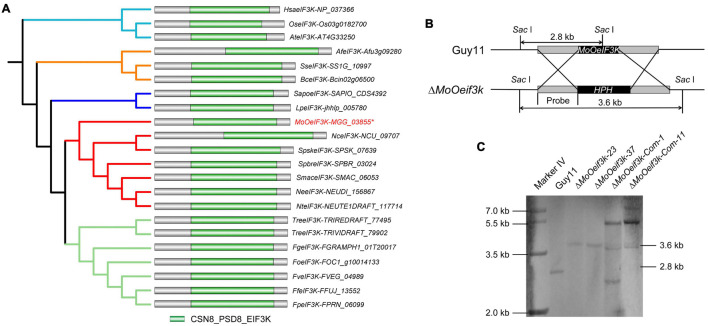
Inter-kingdom phylogeny, domain architecture, and genetic manipulation of *MoOeIF3K* in *Magnaporthe oryzae Oryza* (*MoO)*. Panel **(A)** shows maximum likelihood phylogenetic tree and domain architecture of eukaryotic translation initiation factor 3 (eIF3) from humans, fungi, and plants. **(B)** Schematic probe-map showing homologous recombination targeted gene disruption strategy used to generate Δ*MoOeif3k* strains. **(C)** Southern blot result showing successful replacement of *MoOeIF3K* in the Δ*MoOeif3k* strains with the hygromycin-resistance gene through a single insertion and a successful reintroduction of *MoOeIF3K* into the Δ*MoOeif3k* strains. Maximum likelihood phylogeny for the respective *eIF3K* amino acid sequences were tested with 1,000 bootstrap replicates.

Amino acid sequences of *Homo sapiens-*, *Fusarium graminearum-*, and *Neurospora discreta*-annotated eIF3k were used to run BLASTp and reverse BLASTp research on the fungi and Oomycetes genomic resources platform (Basenko et al., 2018). Results obtained from these multiple blast searches identified a single copy of an uncharacterized (hypothetical) protein containing CSN8/PSMD8/EIF3K domain, here referred to as (*MoOeIF3k*) in the genome sequence assembly data available for the rice blast fungus. Additional phylogenetic analyses were performed to determine the evolutionary pattern of eIF3ks at both class and kingdom levels using MEGAX, Japan version 10.1.7 (Molecular Evolutionary Genetics Analysis software) (Kumar et al., 2016; Barazesh et al., 2020) and deployed online on the Interactive Tree Of Life (iTOL) platform (Letunic and Bork, 2007) for final polishing. Results from these examinations revealed that within the class Sordariomycetes, *MoOeIF3k* shared a common evolutionary clade with eIF3ks identified in *Sporothrix schenckii*, *Sporothrix brasiliensis*, *Sordaria macrospora k-hell*, *Neurospora tetrasperma*, and *N. discreta*; *MoOeIF3k* is, however, evolutionarily distant from eIF3ks identified in *F. graminearum*, *Fusarium oxysporum*, *Fusarium proliferatum*, *Fusarium verticillioides*, *Fusarium fujikuroi*, *Trichoderma virens*, *Trichoderma reesei*, *Lomentospora prolificans*, and *Scedosporium apiospermum*. Meanwhile, eIF3ks from fungi populations in the class Sordariomycetes clustered into a single heterogeneous clade but are phylogenetically distant from eIF3ks from members in the class Eurotiomycetes (*Aspergillus fumigatus*) and Leotiomycetes (*Botrytis cinerea* and *Sclerotinia sclerotiorum*). We also observed that eIF3ks from Sordariomycetes are phylogenetically distant from plants (*Arabidopsis thaliana* and *Oryza sativa* japonica), mammals (*H. sapiens*), Oomycetes, and other fungi classes, such as Saccharomycetes, Ustilaginomycetes, Glomeromycetes, Blastocladiomycetes, and others, lack eIF3k orthologs in their genomes (Figure 1A).

Additional results acquired from Pfam 32.0 domain profiling analysis (El-Gebali et al., 2019) showed that notwithstanding the within and between class and kingdom level phylogenetic differences recorded among eIF3ks from the selected organisms, all the eIF3k sequences from fungi, plants, and mammals possess the typical CSN8/PSMD8/EIF3K (Figure 1A).

To determine the functional role of MoeIF3k in the development of *M. oryzae Oryza* (*MoO*), we accordingly deployed homologous recombination strategies and generated targeted gene deletion strains (Δ*MoOeif3k*), and complemented the Δ*MoOeif3k* strains by transforming the full-length open reading frame (ORF) *MoOeIF3K* and *MoOeIF3K-GFP* fusion constructs into protoplasts prepared from the Δ*MoOeif3k* strains independently and obtained two lines of complementation strains, Δ*MoOeif3k_Com-1* and Δ*MoOeif3k_Com-11*. Results from Southern blotting assays revealed the successful replacement of the *MoOeIF3K* gene by hygromycin B resistant gene at a single locus. Meanwhile, one of the multiple bands observed in the Southern blotting images obtained for the Δ*MoOeif3k*_Com-1 and Δ*MoOeif3k*_Com-11 strains corresponds to the 3.6 kb band in Δ*MoOeif3k* (Figures 1B,C). The results indicated that the complementation strains obtained in this study resulted from random and multiple insertions of the *MoOeIF3K* and *MoOeIF3K-*GFP constructs at other loci within the genome without displacing the hygromycin B-resistant gene from the Δ*MoOeif3k_Com-1* and Δ*MoOeif3k_Com-11* strains. These results suggest that MoeIF3k is a member of the *MoO* non-essential eIF3 complex and plays a functionally dispensable role in the survival of the rice blast fungus.

### *MoOeIF3k* Is Required for Full Morphological Development of the Rice Blast Fungus

**FIGURE 2:**
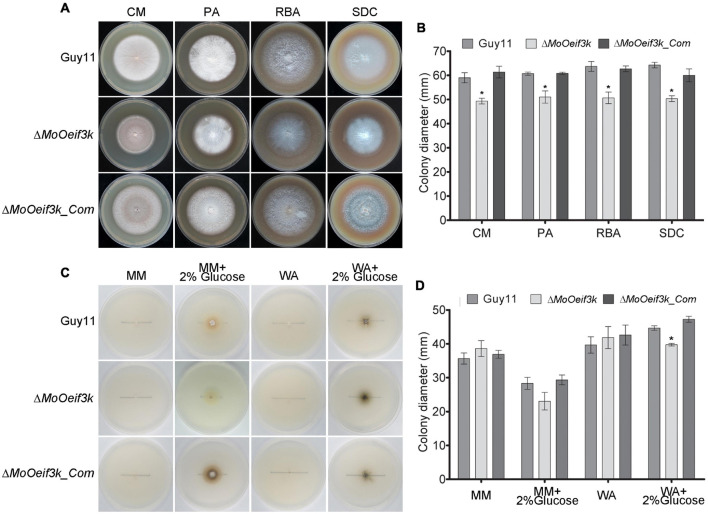
*MoOeIF3k* contributes positively to vegetative growth and survival of *MoO* under starvation conditions. **(A)** Depicts the average vegetative growth of the Δ*MoOeif3k* strains, the complemented strains, and wild-type cultured on nutrient-rich culture media, straw decoction, and corn agar medium (SDC), prune agar (PA), and rice bran agar (RBA) for 10 days. **(B)** Represents the statistical computation of vegetative growth records for Δ*MoOeif3k* strains, the complemented strains, and wild-type cultured on nutrient-rich culture media, straw decoction, and corn agar medium (SDC), prune agar (PA), and rice bran agar (RBA) for 10 days. **(C)** Showed the comparative vegetative growth performance of the Δ*MoOeif3k* strains, complemented strains, and wild-type cultured on nutrient-deficient culture media (MM) and water agar media (WA), meanwhile, MM and WA supplemented with 2% w/v glucose acted as a carbon source. **(D)** Statistical representation of the vegetative growth performance of Δ*MoOeif3k*, the complemented strains, and wild-type strains cultured on MM and WA media with 2% w/v glucose as a carbon source for 10 days. Three independent biological experiments with five replicates each time had consistent results. One-way statistical ANOVA (non-parametric) was performed with GraphPad Prism 6 and Microsoft Excel spreadsheet. Error bars represent the standard deviation, and a single asterisk “*” represents a significant difference in the vegetative growth of the Δ*MoOeif3k* strains and Δ*MoOeif3k_Com* compared with the wild-type (*p* ≤ 0.05).

To gain insights into the contributions of *MoOeIF3k* to the vegetative development of *Mo*O, the Δ*Mo*O*eif3k* strains generated in this study were cultured on different types of nutrient-sufficient and nutrient-deficient culture media. Growth assessment records showed a significant decrease in the vegetative development of Δ*Mo*O*eif3k* strains cultured on different kinds of nutrient-sufficient media, including complete media, straw decoction, and corn agar medium (SDC), prune agar (PA), and rice bran agar (RBA) (Figures 2A,B). Conversely, there was a non-significant but appreciable increase in the vegetative growth of Δ*Mo*O*eif3k* strains grown on nutrient-deficient media, namely, minimum media (MM) and water agar (WA) media. Meanwhile, MM and WA supplemented with 2% w/v glucose as a carbon source triggered a substantial reduction in the growth of Δ*Mo*O*eif3k* strains (Figures 2C,D). Since translation is an energy- and nutrient-intensive cellular process (Doudna and Sarnow, 2007), we averred that the absence of *MoOeIF3k* would not only trigger a reduction in the translational load (protein turn-over) but could also alter the translational dynamics to favor the translation of proteins essential for the mitigation of stress homeostasis in *MoO.* Accordingly, we posited that *MoOeIF3k* acts as a positive and negative regulator of fungal morphogenesis under nutrient-sufficient and deficient conditions, respectively, through direct or indirect translational regulation of proteins associated with morphological development in filamentous fungus under nutrient-specific conditions.

### Contributions of MoeIF3k to Multiple Stress Tolerance of *Magnaporthe oryzae Oryzae*

**FIGURE 3:**
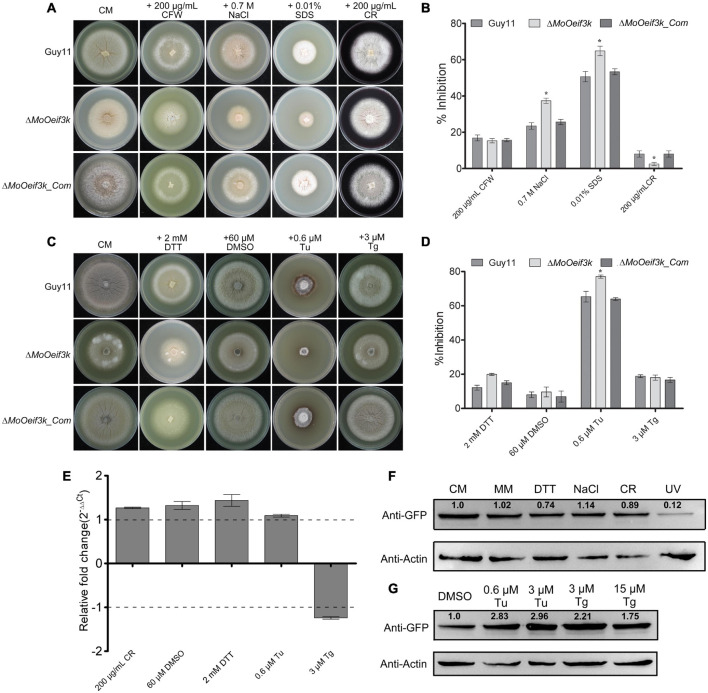
Δ*MoOeif3k* strains displayed differential responses to multiple reductive (ER) stress-inducing agents. **(A)** Growth response of the Δ*MoOeif3k* strains, complemented strains, and wild-type cultured on CM supplemented with either 200 μg/ml Calcofluor White (CFW), 2 mM NaCl, 0.01% SDS, or 200 μg/ml Congo red (CR) as oxidative and ionic stress-inducing osmolytes. **(B)** Statistical representation of the inhibitory effects of oxidative and ionic stress-inducing osmolytes on the vegetative development of the Δ*MoOeif3k* strains, complemented strains, and the wild-type strain. **(C)** Portrays the vegetative growth of the Δ*MoOeif3k*, Δ*MoOeif3k_Com*, and wild-type strains CM supplemented with 2 mM dithiothreitol (DTT), 60 μM dimethyl sulfoxide (DMSO), 0.6 μM tunicamycin (Tu), and 3 μM Tg thapsigargin (Tg) independently as ER-stress inducing agents. **(D)** Statistical representation of the inhibitory effects of different ER-stress inducing agents on the vegetative growth of the Δ*MoOeif3k*, Δ*MoOeif3k*_*Com*, and wild-type strains. Panel **(E)** shows the *in vivo* expression pattern of the putative *MoOeIF3K* gene in the wild-type strain challenged with 2 mM DTT, 60 μM DMSO, 0.6 μM Tu, and 3 μM Tg for 2 h. Real-time quantitative PCR (qRT-PCR) data were computed with the Microsoft Excel spreadsheet and GraphPad Prism 6. Error bars represent mean ± SD, and a single asterisk “*” represents a significant increase or reduction in folds expression of the *MoOeIF3K* gene in the *MoO* strains treated with respective ER-stress-inducing agents compared with the control cultured on either CM (for DTT) or DMSO (for Tu and Tg). Consistent values were obtained with three independent biological replications and three technical replicates for each independent qRT-PCR experiment. Panel **(F–G)** shows the protein level assessment pattern of *MoOeIF3k* in *MoOeIF3k*-GFP strains challenged with DTT, 60 μM DMSO, 0.6 μM Tu, and 3 μM Tg for 2 h. Intensity of *MoOeIF3k* under the individual treatment was evaluated using the following algorithm: [intensity of *MoOeIF3k*-GFP protein treatment/(intensity of actin for treatment × normalize intensity for control)]. Normalized intensity for control group = intensity of *MoOeIF3k*-GFP protein of control/intensity of actin for control. Inhibition data were generated from three independent biological experiments with five technical replicates each time. One-way statistical analysis of variance (ANOVA) (non-parametric) was carried out with GraphPad Prism 6 and Microsoft Excel spreadsheets. Error bars represent the SD. Inhibition rate = (the diameter of untreated strain – the diameter of treated strain)/(the diameter of untreated strain) × 100%. Single and double asterisks represent significant differences (*p* ≤ 0.05 and *p* ≤ 0.02), respectively.

In *C. elegans*, eIF3k functions as a negative regulator of longevity and stress tolerance (Cattie et al., 2016). Therefore, to examine the impact of *MoOeFI3K* gene deletion on the response of the rice blast fungus to oxidative and reductive stress, the Δ*MoOeif3k* strains, along with the complementation and wild-type strains, were cultured on CM supplemented independently with Calcofluor White (CFW), NaCl, SDS, and Congo red (CR) as oxidative (Just and Arendshorst, 2003; Ding et al., 2014; Messina et al., 2014; Khare et al., 2015) stress-inducing agents and DTT, tunicamycin (Tu), and thapsigargin (Tg) as reductive stress-inducing agents (Grant, 2002; De Minicis et al., 2012; Korge et al., 2015; Arrieta et al., 2020; Ma et al., 2020). Results obtained from stress-responsive bioassays showed that the growth Δ*MoOeif3k* strains on complete media (CM) supplemented with oxidative stress-inducing agents, such as sodium dodecyl sulfate (SDS) and NaCl, were significantly inhibited. We observed that the Δ*MoOeif3k* strains were more resistant to CR than the wild-type and complementation strains (Figures 3A,B). The growth of the individual strains on MM supplemented with oxidative and reductive (ER) stress-inducing osmolytes yielded a similar sensitivity response (Supplementary Figures 1A,B). The Δ*MoOeif3k* strains are significantly sensitive to Tu but immune to DTT and Tg, as well as the mock control (DMSO) (Figures 3C,D and Supplementary Figures 1C,D). Meanwhile, results obtained from temperature sensitivity assays showed that the Δ*MoOeif3k* strains displayed almost stable vegetative growth characteristics as the wild-type and complementation strains (Supplementary Figures 2A,B).

Further quantitative PCR (qPCR) evaluation of the expression pattern of the *MoOeFI3K* gene during oxidative and reductive stress response of the rice blast showed that the exposure of *MoO* to different stress-inducing conditions did substantially alter the transcription pattern of *MoOeFI3K* (Figure 3E). However, Western blotting bio-assays revealed a reduction in the expression level of *MoOeIF3k* exclusively during DTT, CR, and UV-induced stress conditions (Figure 3F). On the contrary, we demonstrated that the exposure of *MoO* to Tu- and Tg-induced ER stress (reductive stress) enhanced the accumulation of *MoOeIF3k* (Figure 3G). Meanwhile, we also observed that the deletion of *MoOeIF3K* triggered a slight but non-significant increase in the expression pattern of other MoOeIF3 subunits, especially *MoOeIF3D* and *MoOeIF3H* (Supplementary Figure 3). We subsequently inferred that *MoOeIF3k* contributes significantly to the regulation of osmolyte-specific stress tolerance of *MoO*.

### MoeIF3k Negatively Regulates Ribosomal RNA and Total Protein Turnover in *Magnaporthe oryzae Oryzae*

To unravel the impact of *MoOeIF3K* gene deletion on translation and post-translational processes, we performed a comparative quantification of total ribosomal RNAs (rRNAs) and total protein turnover between the wild-type and defective strains. About 80% of cellular RNAs are rRNAs. Therefore, an rRNA quantification analysis was performed by measuring the concentration of total RNAs extracted from the individual strains and used to compute the approximate total rRNA content using the algorithm (Estimated total rRNA = Concentration of total RNA × 0.8). A comparative analysis of estimated total rRNA level between the wild-type and Δ*MoOeif3k* strains showed that targeted disruption of the *MoOeIF3K* subunit triggered a substantial increase in rRNA generation in the defective strains (Table 1).

**TABLE 1:** Quantitative assessment of the impact of targeted deletion of *MoOeIF3K* gene on the level of cellular ribosomal ribonucleic acid (rRNA) and protein synthesis.

Strain	Total concentration of RNA (ng/μ L)	Estimated total rRNA level (ng/μ L)	OD_260/280_	Concentration of total protein (mg/mL)
Guy11	1216.2	973.0	2.21	9.528
Guy11	1140.0	912.0	2.21	9.528
Guy11	1156.7	925.4	2.24	9.368
D*MoOeif3k*	2333.1	1866.5	2.23	10.968
D*MoOeif3k*	2041.7	1633.4	2.27	11.288
D*MoOeif3k*	2092.0	1673.6	2.19	11.888

Also, to ascertain the significance of the substantial increase in rRNA level on total protein output in the Δ*MoOeif3k* strains, we performed a bicinchoninic acid (BCA) assay-mediated comparative quantification of total protein turnover between the wild-type and Δ*MoOeif3k* strains. The examinations revealed an increased total protein level in the Δ*MoOeif3k* strains compared with the wild-type (Table 1). Given the concomitant increase in total rRNA and protein level in the *MoOeIF3K*-defective strain, we concluded that *MoOeIF3K* likely influences multiple developmental processes in the rice blast fungus through the regulation of protein synthesis.

### *MoOeIF3k* Differentially Modulates the Progression of Asexual and Sexual Reproduction in *Magnaporthe oryzae Oryzae*

**FIGURE 4:**
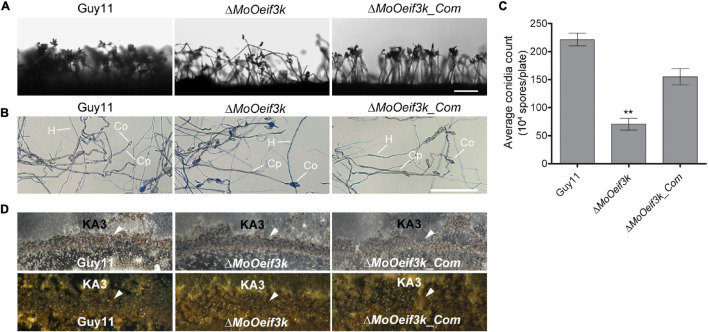
*MoOeIF3k* has a role indispensable in asexual reproduction but dispensable in the progression of sexual reproduction in *MoO*. Panel **(A)** displays conidiophore and the level of asexual spores produced by Δ*MoOeif3k* strains, Δ*MoOeif3k_Com* strains, and the wild-type strain cultured under the same growth conditions. **(B)** The micrograph obtained distinctive staining of vegetative hyphae with lactophenol cotton blue solution to differentiate conidiophores from vegetative hyphae and conidia. The gray-white depict conidiophore (Cp), the vegetative hyphae (H), and conidia (Co) stain blue coloration. **(C)** Comparative statistical presentation of asexual sporulation characteristics of the Δ*MoOeif3k* strains, Δ*MoOeif3k_Com* strains, and the wild-type strain cultured under the same growth conditions. **(D)** Sexual reproduction experiments showed that the wild-type Guy11 strain (mating type 1-1) × KA3 strain (mating type 1-2) produced numerous perithecia in contact zones between the compatible mating types strains on oatmeal agar medium; the level perithecia produced form the crossing of the Δ*MoOeif3k*, and Δ*MoOeif3k_Com* strains with KA3 strain were similar to the level observed in the wild-type strain. The arrow head indicates the perithecia in the contact zones, and scale bar: 50 μm. Double asterisks “**” represent a statistical significant difference of p ≤ 0.01.

Sporulation plays a significant role in both the perpetuation and dissemination of the rice blast disease. Unique proteins or pathways drive asexual and sexual reproduction in fungi (Cvitanich and Judelson, 2003; Schoustra et al., 2010; Park and Yu, 2012; Son et al., 2014). To unravel the potential contributions of *MoOeIF3k* to asexual reproduction in the rice blast fungus, we monitored conidiophore formation. We also performed a comparative assessment of the number of conidia produced by the Δ*MoOeif3k*, complementation, and wild-type strains cultured on RBA to induce the production of asexual spores. We observed that the deletion of *MoOeIF3K* attenuated conidiophore development and significantly suppressed conidia production in the Δ*MoOeif3k* strains (Figures 4A–C).

Additional results obtained from mating assays performed in this study to examine the influence of *MoOeIF3K* on sexual reproduction in *MoO* revealed that *MoOeIF3K* modulates the progression of sexual reproduction in the rice blast fungus independent of *MoOeIF3k* function. Hence, sexual sporulation characteristics displayed by the Δ*MoOeif3k* strains were indistinguishable from those of the wild-type and complementation strains (Figure 4D). These results suggest that *MoOeIF3k* likely influences asexual reproduction in *MoO* by modulating cellular processes or pathways independent of sexual reproduction in filamentous fungi.

### Targeted Gene Deletion of *MoOeIF3K* Attenuates the Virulence of *Magnaporthe oryzae Oryzae*

**FIGURE 5:**
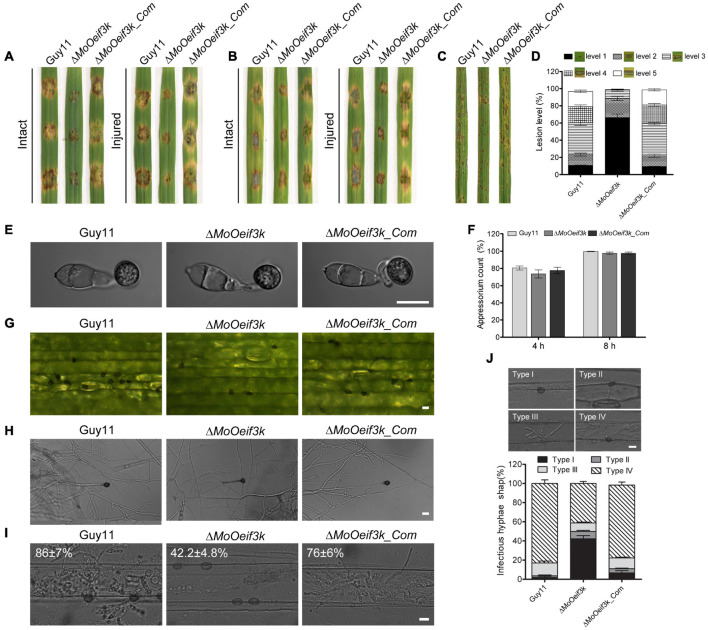
Impact of *MoOeIF3K* gene deletion on virulence, appressorium morphogenesis, and penetration characteristics of *MoO*. **(A)** Hyphae-mediated infection competence of the Δ*MoOeif3k* strains on intact and injured barley leaves compared with that of the complementation and wild-type strains. **(B)** Portrays conidia-mediated infection competence of the Δ*MoOeif3k* strains on intact and injured barley leaves compared with that of the complementation and wild-type strains. Panel **(C)** displays the virulence capabilities of the Δ*MoOeif3k* strains, Δ*MoOeif3k_Com*, and the wild-type strains on 2-week old susceptible CO39 rice seedlings inoculated with spore suspensions. **(D)** Statistical representation of results recorded from the semi-quantitative evaluation of pathogenicity and virulence characteristics of the individual strains. Almost consistent results obtained from three biological infection assays with five technical replicates were used for the statistical analyses. In each replicate, 200 lesions were counted. Thus, total number of lesions (*n*) = 1,800. **(E)** Comparative appressorium morphology of conidia produced by the Δ*MoOeif3k*, Δ*MoOeif3k_Com*, and wild-type strains. **(F)** Bar graph shows statistical computation of records obtained from microscopy examination of the appressorium formed by conidia from the wild-type and Δ*MoOeif3k* strains on hydrophobic coverslips at 4 and 8 hpi. **(G)** Micrograph shows a reduction in the hyphae tip appressorium-like structures produced by the Δ*MoOeif3k* strains inoculated on excised barley leaves compared with the complementation and wild-type strains. **(H)** Micrograph shows the morphology of hyphae tip appressorium-like structures produced by the Δ*MoOeif3k* strain-inoculated hydrophobic coverslips compared with the complementation and wild-type strains. **(I)** Histopathology micrograph shows the comparative penetration and colonization efficiency of the Δ*MoOeif3k*, Δ*MoOeif3k_Com*, and wild-type strains 48 h post inoculation of barley leaves with spore suspensions. Scale bar: 10 μm. Panel **(J)** shows comparative quantitative analyses of invasive development of the individual strains classified into four types: type I (appressorium), type II (primary invasive hyphae), type III (invasive hyphae), and type IV (with extensive hyphal growth). Statistical evaluations were conducted with almost consistent values obtained from three biological experiments with three technical replicates each time. For each replicate, a total of 100 infection sites (thus *n* = 300) were scanned under a microscope. One-way statistical ANOVAs (non-parametric) were performed with GraphPad Prism 6 and Microsoft Excel spreadsheets. Error bars represent the standard deviation.

To examine the pathogenicity or virulence efficiency of hyphae and asexual spores produced by Δ*MoOeif3k*, we inoculated intact and injured leaves of the “golden promise” barely cultivar with mycelia harvested from the Δ*MoOeif3k* strains, along with the complementation and wild-type strains. At the same time, barley leaves were drop-inoculated with spores in suspension, and leaves of the susceptible rice seedlings (CO39) were spray-inoculated with spores from the individual strains. Records obtained from these infection assays showed that the deletion of *MoOeIF3K* severely compromised both mycelia, and that conidia mediated the development of blast symptoms on both intact and injured leaf tissues (Figures 5A–C). Additional results obtained from a semi-quantitative assessment of virulence characteristics of the individual strains revealed a remarkable reduction in the severity of blast infections caused by the Δ*MoOeif3k* strains on the blast-susceptible CO39 rice seedlings (Figure 5D).

Appressorium and hyphae tip appressorium-like structures are the fundamental infectious structures that support the development of conidia- and hyphae-mediated blast infections, respectively. To identify some of the possible factors accounting for attenuation in the virulence of the Δ*MoOeif3k* strains, conidia produced by the Δ*MoOeif3k* strains, the wild-type strain, and the Δ*MoOeif3k_Com* strain were inoculated on appressorium-inducing hydrophobic coverslips to assay the germination and appressorium formation characteristics of the Δ*MoOeif3k* strain relative to the wild-type and complementation strains *in vitro*. The formation of hyphae tip appressorium-like structures was assayed by inoculating mycelium from the Δ*MoOeif3k* strain, the wild-type strain, and the Δ*MoOeif3k*_*Com* strain on the barley leaves and on hydrophobic coverslips (Lin et al., 2019). Corresponding results obtained from microscopy examinations showed that the morphology of the appressorium produced by the Δ*MoOeif3k* strains was comparable to those produced by the wild-type strain (Figures 5E–H).

However, results obtained from histopathological examinations showed that targeted gene disruption of *MoOeIF3K* severely compromised both penetration and colonization efficiencies of Δ*MoOeif3k* (Figures 5I,J). We inferred that virulence defects displayed by the Δ*MoOeif3k* strains are not due to distortions in the morphological integrity of appressoria and appressorium-like structure formed by the Δ*MoOeif3k* strains.

### Deletion of *MoOeIF3K* Triggered a Significant Reduction in the Mobilization of Appressorium Turgor

**FIGURE 6:**
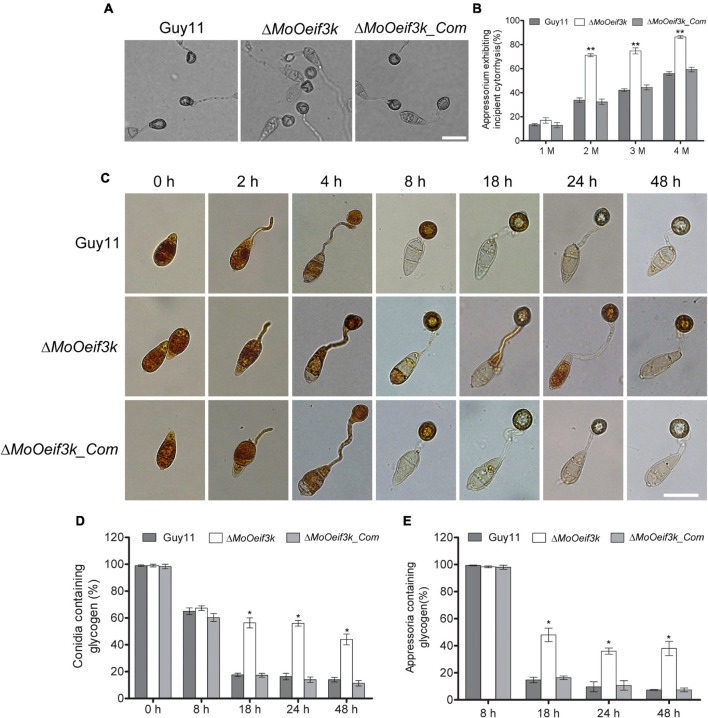
Deletion of *MoOeIF3K* compromised the integrity of appressorium turgor, transport, and degradation of glycogen during appressorium morphogenesis in *MoO*. Panel **(A)** shows incipient cytorrhysis assays carried out to measure appressorium turgor for the Δ*MoOeif3k*, Δ*MoOeif3k_Com*, and wild-type strains inoculated on appressorium-inducing hydrophobic coverslips for 8 h. The appressoria were treated with different concentrations (1, 2, 3, and 4 M) of glycerol solutions. Appressorium collapse was observed and counted under an Olympus DP80 light microscope. Scale bar: 20 μm. **(B)** Statistical representation of proportion-collapsed appressorium recorded for the individual strains under the respective treatments. Statistical analyses were performed with data obtained from three biological replications with three technical replicates each time. For each biological replicate, a total of 100 appressoria were counted (*n* = 100 × 3). Asterisks “*” represent a statistically significant difference of *p* ≤ 0.05. **(C)** Micrograph exhibits glycogen mobilization, transport from conidium to appressorium, and degradation defects associated with the Δ*MoOeif3k* strains during appressorium formation. Conidia harvested from Δ*MoOeif3k*, wild-type, and complemented strains were inoculated on hydrophobic coverslips to induce germination appressorium formation. The drop of water was replaced with an iodine solution at 0, 2, 4, 8, 16, 24, and 48 hpi and was allowed to stand for 2 min. Glycogen dynamics in the individual strains at different time intervals during germination and appressorium formation were observed under a light microscope. Iodine stains glycogen to give yellowish-brown colorations under a microscope. Panels **(D,E)** display statistical evaluation of glycogen level in aberrant conidia, germinated conidia, and germinated conidia with appressorium at different stages of pathogenic development of the individual strains. For each biological replicate, a total of 100 appressoria were counted (n = 100 × 3). Asterisks “*” represent a statistically significant difference of *p* ≤ 0.05 while double asterisks “**” represent a statistically significant difference of *p* ≤ 0.01. Scale bar: 10 μm.

In an attempt to evaluate the functionality of appressorium and appressorium-like structures formed by the Δ*MoOeif3k* strains, we instituted glycerol-mediated incipient cytorrhysis assays to monitor the accumulation of turgor pressure in the appressorium and appressorium-like structures. Findings from these investigations revealed that targeted gene disruption of *MoOeIF3K* compromised appressorium and appressorium-like structure turgor (Figures 6A,B).

We monitored the integrity of appressorium produced by the individual strains by independently staining with iodine and BODIPY^TM^ 493/503 at different stages to observe the mobilization and accumulation of glycogen and lipid bodies, respectively.

Furthermore, we demonstrated that the deletion of *MoOeIF3K* substantially delayed the timely mobilization of glycogen to the appressorium of Δ*MoOeif3k* strains 8, 18, and 24 h post inoculation (hpi) relative to the wild-type and complementation strains (Figures 6C–E). Interestingly, we noticed that the deletion of *MoOeIF3K* had no adverse effect on the mobilization of lipid bodies during appressorium morphogenesis in *MoO*; hence, lipid body mobilization features displayed by the Δ*MoOeif3k* strains were comparable with those displayed by the wild-type and complementation strains (Supplementary Figure 4).

Accordingly, we concluded that *MoOeIF3k* likely contributes to appressorium integrity through selective translational regulation of proteins associated with turgor development and glycogen mobilization.

### Subcellular Localization of MoeIF3k in *Magnaporthe oryzae Oryzae*

**FIGURE 7:**
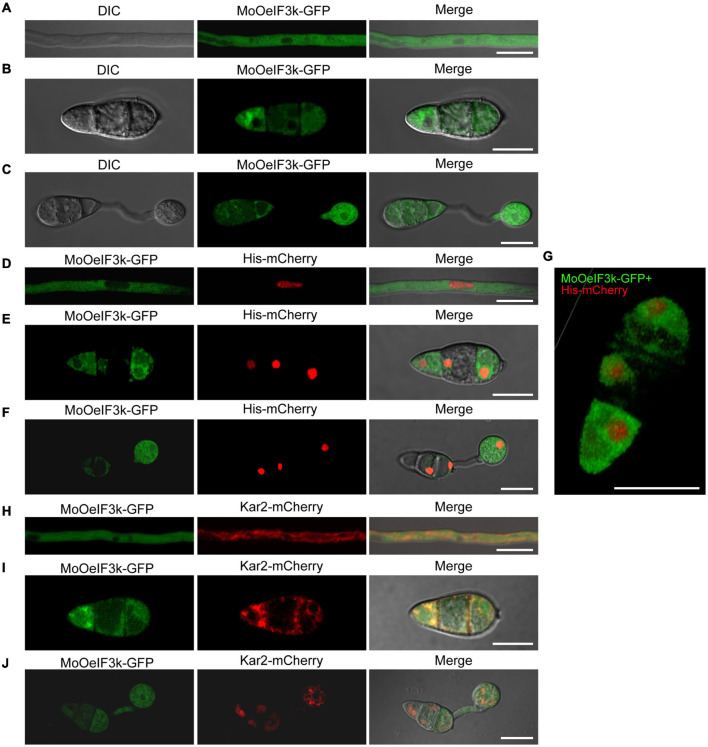
*MoOeIF3k* displayed filamentous cytoplasmic localization patterns during both physiological and pathogenic development of in *MoO*. **(A)** Confirmation of localization of *MoOeIF3k* in the vegetative hyphae. **(B)** Localization of *MoOeIF3k* in the conidia. Panel **(C)** displays the localization of *MoOeIF3k* during conidia germination and appressorium formation. **(D–F)** Co-localization micrograph confirms the exclusion of *MoOeIF3k* from the nuclear as it does co-localize with the Histone-mChery (His-mCherry) nuclear marker. **(G)** Three-dimensional (3D) micrograph shows the unitary localization pattern of *MoOeIF3k*-GFP and the spatial co-localization of *MoOeIF3k*-GFP and His-mCherry in the asexual spore. Panels **(H–J)** show the co-localization of *MoOeIF3k*-GFP with ER-specific marker Kar2-mChery during vegetative and pathogenic development of the rice blast fungus. Localization and co-localization assays were performed with a NikonA1 confocal microscope. Scale bar: 10 μm.

From the fundamental knowledge that translation events occur on either free ribosomes within the cytosol or ER-associated ribosomes, we envisage that eIFs could localize to either the cytoplasmic region or the ER. However, results obtained from previous subcellular localization assays showed that eIFs, such as eIF3k, localize to multiple cellular compartments, such as nuclear (Ahmed, 2001; Marchione et al., 2013). Therefore, to ascertain the subcellular localization of *MoOeIF3k* at different developmental stages of *MoO*, we accordingly performed microscopy assays using hyphae, conidia, and appressorium obtained from the Δ*MoOeif3k_Com* strain harboring a *MoOeIF3k*-GFP fusion construct. The *MoOeIF3k*-GFP displayed a filamentous cytoplasmic localization pattern with intense GFP fluorescence signals around the perinuclear region (Figures 7A–C). Meanwhile, *MoOeIF3k*-GFP and Histone-mCherry (His-mCherry) fusion co-localization assays confirmed that *MoOeIF3k*-GFP localizes to both the cytoplasm, and the perinuclear region at all developmental stages of the rice blast fungus but not the nuclear (Figures 7D–F). At the same time, we demonstrated that *MoOeIF3k*-GFP co-localized with the ER marker (Kar2-mChery) to the ER at all developmental stages (Figures 7G–J). These results indicated that MoeIF3k mediates the translation of mRNAs in the cytoplasm and the ER in *MoO*.

### *MoOeIF3k* Possibly Facilitates a Cross-Talk Between Transcriptional and Translational Regulatory Machinery Pathogen–Host Interaction

**FIGURE 8:**
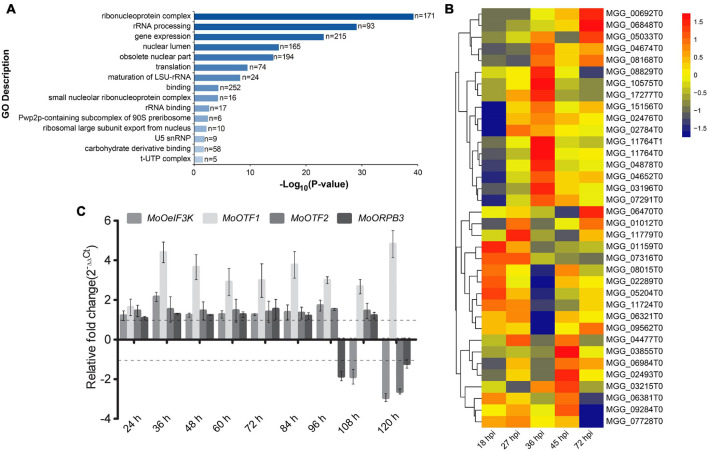
Putative *MoOeIF3k* interaction partners and the expression pattern of *MoOeIF3K* and transcriptional regulatory mechanisms during pathogen–host interaction. **(A)** GO terms and pathway enrichment map provide insights into biological process, molecular function, and cellular processes influenced by *MoOeIF3k* putative interacting proteins in the rice blast fungus. **(B)** Heat map displays the expression pattern of *MoOeIF3K* and exclusive interacting putative transcription factors identified in the *MoOeif3k*-GFP immune-complex at different stages of *MoO-*host (rice) interaction. **(C)** Confirmatory RT-qPCR assays showing the three putative TFs that were co-induced along with *MoOeif3K* 45 hpi. Data used in RT-qPCR analyses were obtained from three biological experiments with three technical repeats each. The expression pattern of the individual genes 12 hpi was adopted as the reference stage.

To identify a possible cross-talk between *MoOeIF3k* and transcriptional regulation machinery during pathogen–host interaction (PHI), we extracted protein Guy 11 strains harboring *MoOeIF3k*-GFP fusion constructs and control Guy 11 strains containing the vehicle vector with GFP (RG7) for co-immunoprecipitation (Co-IP) assays using GFP-Trap beads. Comparative co-IP analyses revealed a total of 531 *MoOeIF3K*-GFP co-immunoprecipitation protein complexes. Furthermore, Gene Ontology (GO) and pathway enrichment analyses showed that putative *MoOeIF3k*-interacting proteins recovered from the *MoOeIF3k*-GFP immune-complex predominantly consist of proteins associated with, ribonucleoprotein complex (171 proteins), rRNA processing (93 proteins), and regulation of gene expression (215 proteins) (Figure 8A). Putative *MoOeIF3k* interacting proteins associated with the regulation of gene expression include 34 transcription factors (TFs) (Supplementary Table 2) and other components of the eIF3-complex.

Also, we examined the likely co-regulation of MoeIF3k on the 34 TFs recovered from the *MoOeIF3k*-GFP immune-complex during PHI by monitoring the expression pattern of *MoOeIF3K* along with the 34 TFs in multiple infection-stage transcriptomic data generated by Jeon et al. (2020) and deposited at public repository database NCBI Sequence Read Archive and assigned with accession numbers SRR8259726 to SRR8259732. Results obtained from these examinations revealed a partial co-regulation and induction in the expression of *MoOeIF3K* and putative TFs encoded by DNA-directed *MGG_03215* (*MoORPB3*), *MGG_02493* (*MoOTF2*), and *MGG_07728* (*MoOTF1*) (Figure 8B). Additional RT-qPCR assays confirmed a partial correlation between the expression of *MoOeIF3K*, *MoORPB3*, *MoOTF2*, and *MoOTF1* during PHI (Figure 8C). These observations suggest a possible co-regulation cross-talk between transcriptional and translational regulatory machinery during the progression of PHI.

### Interaction Relationship Between MoeIF3k and Subunits of the CSN Complex in *Magnaporthe oryzae Oryzae*

**FIGURE 9:**
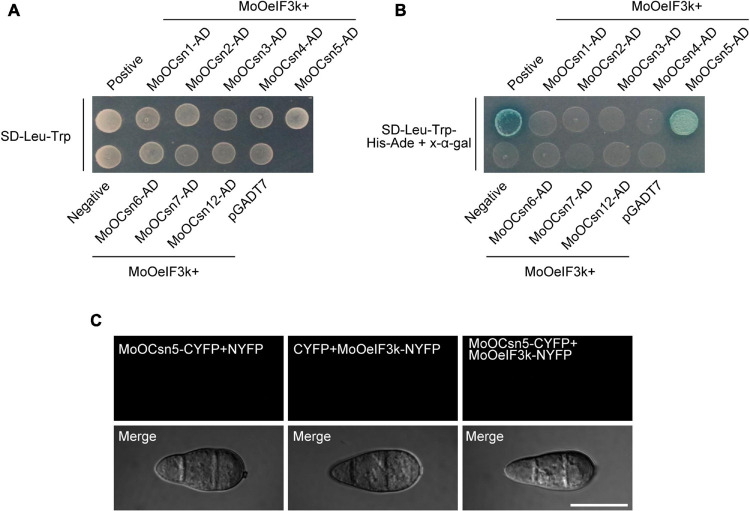
*In vitro* interaction network between *MoOeIF3k* and subunits on the CSN complex. **(A)** Yeast-two-hybrid assay of the growth yeast strains harboring *MoOeIF3k*-BD-paired MoOCsn-AD constructs of individual subunits of the CSN complex on synthetic defined (SD) minimal yeast media plates supplemented with Trp-Leu as a measure of interaction between the paired subunits. Panel **(B)** shows the growth of yeast transformants expressing the *MoOeIF3k*-BD and MoOCsn-AD constructs on SD supplemented with Trp-Leu-His-Ade + X-α-gal. **(C)** Micrograph portrays the *in vivo* interaction pattern between *MoOeIF3k* and MoOCsn5 in MoO.

Also, co-IP-mediated interactome analyses revealed MoOCsn4 as one of the putative interactors of *MoOeIF3k* (Supplementary Table 3). Since earlier domain search analyses identified a conserved CSN8_PSD8_eIF3K motif as an inseparable domain across corresponding orthologs from animals, fungi, and plants species sampled for phylogenetic analyses conducted in this study, we deployed yeast-two-hybrid (Y2H) assays to examine the possible existence of direct/physical interaction of *MoOeIF3K* and subunits of the COP9/CSN-signalosome complex in the rice blast fungus and also to partly dispel any likely assumptions that might seek to project *MoOeIF3K* as a subunit of the CSN complex. Interestingly, while no physical interaction was observed between *MoOeIF3k*-BD vs. MoOCsn1-AD, *MoOeIF3k*-BD vs. MoOCsn2-AD, *MoOeIF3k*-BD vs. MoOCsn3-AD, *MoOeIF3k*-BD vs. MoOCsn4-AD, *MoOeIF3k*-BD vs. MoOCsn6-AD, *MoOeIF3k*-BD vs. MoOCsn7-AD, and *MoOeIF3k*-BD vs. MoOCsn12-AD, our investigations revealed the existence of a possible physical interaction between *MoOeIF3k*-BD vs. MoOCsn5-AD (Figures 9A,B). However, previous studies have shown that Csn5-AD interacts directly with the GAL4 DNA-binding domain (DBD) to yield false-positive results in GAL4-mediated Y2H assays (Nordgård et al., 2001). Hence, to confirm whether the interaction observed between *MoOeIF3k*-BD vs. MoOCsn5-AD emanated from false binding between the GAL4-DBD domain and MoOCsn5-AD, about 10 μl of AH109 yeast strains harboring the empty GAL4-DBD (pGBKT7) and MoOCsn5-AD constructs were grown on SD-Leu-Trp-His-Ade media with surface fortified with X-α-gal (Supplementary Figure 5). Results from these examinations confirm the interaction between the DB domain-containing vehicle pGBKT7 and MoOCsn5-AD. Meanwhile, no interaction was observed between MoOCsn5-AD and pGADT7 when strains harboring MoOCsn5-AD pGADT7 constructs were cultured on SD-Leu-Trp-His-Ade media with the surface fortified with X-α-gal. Additional results from bimolecular fluorescence complementation (BiFC) analyses performed in this study further confirmed the absence of direct interaction between *MoOeIF3k* and MoOCsn5 (either MoOCsn5-CYFP + *MoOeIF3k*-NYFP, MoOCsn5-CYFP + NYFP, or CYFP + *MoOeIF3k*-NYFP) *in vivo* (Figure 9C). The absence of physical interaction between *MoOeIF3K* and the eight subunits of the CSN complex in *MoO* coupled with the high sequence similarity of *MoOeIF3K* with *eIF3K* orthologs from other organisms further confirmed *MoOeIF3k* as a putative member of the eIF3 complex.

## Discussion

The eIF3 is a conserved multi-protein complex that consists of 5–13 subunits (Sun et al., 2011; Sadato et al., 2018). The eIF3 complex comprises two sub-complexes, namely, essential and non-essential complexes. Collectively, the eIF3 complex (essential and non-essential) plays a fundamental role in facilitating the synthesis of structurally and functionally diverse groups of proteins by acting in association with other initiation factors to stimulate mRNA binding methionyl-tRNAi to the 40S ribosome (Kim et al., 2007; Choudhuri et al., 2010; Hinnebusch, 2011). While the number of subunits that constitute the octameric essential eIF3 sub-complex is conserved among eukaryotes, the number of subunits constituting the non-essential sub-complex varies between kingdoms (Browning and Bailey-Serres, 2015; Zeman et al., 2019). A blast search showed that the eIF3 complex in *MoO* comprises 12 subunits and includes a hypothetical protein containing the eIF3k domain (*MoOeIF3k*). Previous evolutionary and biochemical studies have shown that some subunits of eIF3 assume a regulatory role and are not essential for eIF3 holo-complex functionality (Choudhuri et al., 2010; Hinnebusch, 2011; Shah et al., 2016). We demonstrated that *MoOeIF3k* shared closer phylogenetic ties with pathogenic biotrophic and hemi-biotrophic fungi pathogens, such as *S. schenckii* but evolutionary distant from necrotrophic fungi pathogens and plants, such as *S. sclerotiorum*, *B. cinerea*, and *A. thaliana*. We also showed that some taxonomic classes within the fungi kingdom, such as Saccharomycetes, Ustilaginomycetes, Glomeromycetes, and Blastocladiomycetes lack the eIF3k subunit. The successfully targeted replacement of the non-universally conserved eIF3k complex subunit of the eIF3 complex in *MoO* suggests that eIF3k and possibly other members of the non-essential eIF3 sub-complex could be prone to genetic manipulation without lethal consequences. The absence of the eIF3k subunit in fungi species from selected taxonomic classes, such as Saccharomycetes, Ustilaginomycetes, Glomeromycetes, and Blastocladiomycetes coupled with the fact that majority of species in these classes do not require appressorium to initiate infection, or are non-pathogenic, subsequently informed two sets of reasoning.

First, we reasoned that *MoOeIF3k* likely represents one of the regulatory subunits of the eIF3k complex that conditionally regulates the translational initiation of proteins required pathogenic development and other secondary characteristics, such as stress tolerance, virulence, sporulation, and vegetative development of the rice blast fungus. On the other hand, we speculated that *MoOeIF3k* could play an overlapping or complementary role with different subunits within the eIF3 complex, rendering it partially redundant and dispensable in the physiological or pathological development of *MoO*. The translation of mRNAs that code for proteins associated with cell proliferation is initiated explicitly by the eIF3 complex (Lee et al., 2015; Shirokikh and Preiss, 2018). In *Neurospora crassa*, the deletion of *eIF3K* had no adverse effects on the vegetative growth of Δ*Nceif3k* strains (Smith et al., 2013). Although targeted disruption of *eIF3K* (*CeeIF3K*) in *C. elegans* caused a significant reduction in brood size and enhanced egg-laying capacity, conversely, it improved the lifespan of the defective strains (Cattie et al., 2016). Similar to *CeeIF3K*, the phenotypic assessment of growth characteristics of Δ*MoOeif3k* revealed a significant impairment in the vegetative growth of Δ*MoOeif3k* on different types of nutrient-rich culture media. These findings showed that the non-universally conserved eIF3k subunit of the eIF3 complex plays diverse roles in filamentous fungi. The survival of living organisms under starvation conditions is expenditure on stored cellular nutrients and energy (McCue, 2010; Rabinowitz and White, 2010; Vettor et al., 2020). Surprisingly, our investigations revealed a non-significant but substantial increase in the vegetation of Δ*MoOeif3k* strains under nutrient-deficient conditions compared with the wild-type. Since translational regulation of protein synthesis under starvation conditions is one of the most energy-efficient mechanisms that allow eukaryotic organisms to rapidly and selectively promote the translation of proteins that vitally support their survival under multiple stress conditions, such as starvation, we conclude that *MoOeIF3k* modulates the economic utilization of stored cellular nutrients to promote the survival of the rice blast fungus by acting as a negative regulator of vegetative growth under starvation conditions. A repertoire of stress factors impacts negatively on the proper operations of the ER and undermine the proper folding or refolding of misfolded proteins and the timely degradation of misfolded proteins, resulting in the activation of unfolded protein response (UPR) with deleterious consequences for eukaryotic cells (Cattie et al., 2016). One of the strategies deployed by eukaryotic cells to successfully mitigate the harmful effects of ER stress involves placing restrictions on the biosynthesis of “housekeeping” proteins to favor the biosynthesis of proteins that support stress tolerance by the activation of alternative translational initiation regulation mechanisms (Jaud et al., 2020). Studies have shown that the loss of subunits *eIF3K* and *eIF3L* of the eIF3 complex in *C. elegans* enhanced the resistance of corresponding mutants to ER stress (Cattie et al., 2016). The Δ*MoOeif3k* strains exhibited moderate sensitivity to the ER stress-inducing agent DTT, but were significantly inhibited by Tu, NaCl, and SDS (Liu et al., 2007; Cattie et al., 2016; Yoshino et al., 2017). We posited that unlike eIF3k orthologs in *N. crassa* and *C. elegans*, *MoOeIF3k* functions as a positive regulator ER and ionic (oxidative) stress tolerance in *MoO*. Therefore, the deletion of *MoOeIF3K* possibly enhances the tolerance of the resulting mutant strains to ER stress because the absence of *MoOeIF3K* will practically limit the transport of proteins to ER and reduce the buildup of misfolded proteins (minimize UPR). Although the set of ER and oxidative stress-responsive proteins directly or indirectly regulated by the *MoOeIF3k* subunit is still unclear, previous research studies have shown that the cytoplasmic aggregation of protein and the accumulation of misfolded proteins trigger reductive stress and consequently result in cardiomyopathy in humans (Ma et al., 2020). Therefore, we inferred that targeted replacement of the *MoOeIF3K* gene likely minimizes the translation of mRNAs to proteins, thereby decreasing the inherent generation of reductive stress in the cell and probably accounted for the substantial tolerance of the Δ*MoOeif3k* strains to exogenously induce reductive stress.

Also, an increase or unregulated translation and protein biosynthesis, particularly at the ER, often accumulate misfolded proteins in the ER and result in the induction of UPR (Jiang et al., 2015). Here, we demonstrated that the disruption of *MoOeIF3K* substantially enhanced the generation of rRNAs. rRNAs are the predominant structure component of the ribosome and play crucial roles in promoting the progression of protein synthesis by physically enforcing the tRNAs to drive the translation of mRNAs into protein (Lackner et al., 2007; Perry, 2007; Piques et al., 2009). A corresponding acceleration protein synthesis accompanies the increase in rRNA levels in the Δ*MoOeif3k* strains. These observations provide better insights into the differential sensitivity of the *MoOeif3k* strains towards rapid transient ER stress-inducing agent (Tu) on one hand and DTT, which, on the other hand, induces slow but prolong ER stress (Li et al., 2011). Therefore, linking the fundamental differences in stress-inducing attributes of Tu and DTT to the remarkable accelerations in rRNA levels and total protein level in the Δ*MoOeif3k* strains, we deduced that any rapid induction in ER stress (as is the case under Tu treatment) would increase the burden of stress on the ER, which could already be battling with the increase in protein synthesis and its attendant surge in UPR and consequentially render the Δ*MoOeif3k* strains more vulnerable. However, the induction of ER stress slow under DTT treatment allows for the mobilization or activation of other pathways associated with the mitigation of multiple stresses at the ER. Putting together, we positioned that eIF3K and possibly the non-essential eIF3 complex likely mitigate stress tolerance in the rice blast fungus through the regulation of translational initiation and protein synthesis. Additionally, we reasoned that the rice blast fungus deploys *MoOeIF3K* as an alternative negative regulator of wasteful protein synthesis as an adaptation to support survival under harsh conditions.

Meanwhile, the accumulation of high sustained glycerol-mediated appressorium turgor prior to the transportation of stored energy-rich products, such as glycogen, trehalose, and lipids, from the conidial to the appressorium for subsequent degradation, is among critical biochemical events that support appressorium functionality and promotes successful plant infection (Wang et al., 2005). Here, we demonstrated that the deletion of *MoOeIF3k* compromised the virulence of the resulting mutant strains. However, it is unclear whether multiple pathways mediate the transports of conidia contents to the developing appressorium. According to the biochemical characterization of Δ*MoOeif3k* strains, the disruption of *MoOeIF3k* attenuated appressorium turgor and also exclusively suppressed the transportation and degradation of glycogen (glycophagy) but has no adverse effect on the degradation of lipid bodies (lipophagy) during conidial germination and appressorium formation, indicating that the eIF3k subunit contributes positively to the progression of pathogenesis in *MoO* by regulating likely exclusive glycogen-trafficking pathways and by partially enhancing the integrity of appressorium turgor. Also, nutrient limitation, physiological maturity, and harsh environmental conditions are crucial factors that signal the initiation and progression of sporulation in filamentous fungi (Cvitanich and Judelson, 2003; Noble and Andrianopoulos, 2013; Matheis et al., 2017; Wallen and Perlin, 2018). The significant reduction in asexual sporulation characteristics of the Δ*MoOeif3k* strains, coupled with delayed mobilization and degradation of glycogen during appressorium morphogenesis, further affirms the earlier position that *MoOeIF3k* negatively regulates vegetative morphogenesis during nutrient starvation and, hence, positively regulates asexual sporulation. This study provides additional insights into the role of the eIF3k subunit in both the physiological and pathological development of the rice blast fungus. This study also underscored the need to comprehensively evaluate the influence of the remaining subunits of the non-essential eIF3 sub-complex on the physiological and selective biosynthesis of virulence-related (pathogenesis) proteins during PHI.

## Materials and Methods

### Fungal Strains and Culture Conditions

The parental wild-type *MoO* (Guy11) strain used as a background in the generation-targeted gene-replacement mutant strains for the *MoOeIF3K* gene characterized in this study was a gift from Dr. Didier Tharreau (CIRAD, Montpellier, France). Bacteria competent cells used to propagate the constructed plasmids were prepared from *Escherichia coli* strain *DH5*α.

For vegetative growth (either assessment of vegetative growth), the wild-type, mutant, and complementation strains were cultured on a complete medium (CM, 6 g yeast extract, 6 g casein hydrolysate, 10 g sucrose, 20 g agar), PA medium (40 ml prune juice, 2.5 g lactose, 2.5 g sucrose, 1 g yeast extract, 20 g agar, and pH 6.5), SDC (100 g of straw, 40 g of corn powder, and 15 g agar), MM (6 g NaNO_3_, 0.52 g KCl, 0.152 g MgSO_4_⋅7H_2_O, 1.52 g KH_2_PO_4_, 0.01 g VB1, 1 ml microelement, and 20 g agar), WA (20 g agar) at 25°C. For conidiation, the strains were cultured on RBA medium (40 g rice bran, 20 g agar, and pH 6) for 10 days in the dark. The plates were later transferred into an incubator with continuous light for 3 days after removing the vegetative hyphae. For sensitivity assays, the individual strains were cultured on CM plates supplemented with different stress-inducing agents (oxidative stress-inducing agents): 200 μg/ml CFW (F3543; SIGMA, Germany), 0.7 M NaCl, 0.01% sodium dodecyl sulfate, 200 μg/ml Congo red (0379; TAGENE); reductive stress-inducing agents: 2 mM DTT (1758-9030; INALCO, United States), 60 μM DMSO (D8418; SIGMA, Germany), 0.6 μM tunicamycin (A611129; BBI, Germany), and 3 μM thapsigargin (A616759; BBI, Germany).

### Generation of Gene Replacement Mutant and Complementation

Split-marker knockout vectors were constructed and used for the targeted gene replacement of *MoOeIF3K* in *MoO*. To construct split-makers for *MoOeIF3K*, 0.96 kb upstream and 1 kb downstream flanking fragments were amplified with primers eIF3K-AF/AR and eIF3K-BF/BR, respectively. The upstream fragment was fused into the *Kpn*I and *Eco*RI enzyme restriction sites at the upstream half of HPH on pCX62. The downstream fragment was fused into the *Bam*HI and *Xba*I enzyme restriction sites at the downstream half of the HPH on the pCX62 vector by overlap extension PCR cloning (OE-PCR) (Bryksin and Matsumura, 2010). The amplification of PCR products used in the construction of targeted gene replacement vectors was performed using the primer pairs eIF3K-AF + YG/R and HY/F + eIF3K-BR (Supplementary Table 1). The preparation of the *MoO* protoplast and fungal transformation were performed as described by Talbot et al. (1993) and Nakayashiki et al. (2005). The transformants were screened with eIF3K-OF/eIF3K-OR and eIF3K-UF/eIF3K-UR. The knockout candidates were further confirmed with a Southern blotting assay.

To construct a complementation/*MoOeIF3k*-GFP fusion vector, a 3,081-bp fragment, including native promoter and whole ORF sequence without stop codon, was amplified. The product was fused into the *Eco*RI and *Bam*HI enzyme restriction sites upstream of the GFP site on the pKNTG vector. The constructed vector was transformed into the protoplast of the Δ*MoOeif3k* strain. The transformants were screened with PCR using the primer pairs (eIF3k-OF and GFP-R).

### Genomic DNA Isolation

Genomic DNA extraction from the wild-type Guy11, Δ*MoOeif3k*, and complementation strains was performed with the CTAB DNA extraction procedure described by Brandfass and Karlovsky (2008) and Aliyu et al. (2019). Briefly, the fungal strains were cultured in a CM liquid medium for 4 days at 28°C, 110 rpm. Mycelia were harvested and press-dried with sterilized absorbent filter paper, frozen, and ground into powder in liquid nitrogen. The samples were placed into 2-ml Eppendorf tubes containing 1 ml of cold DNA extraction buffer (100 mM Tris–HCl 8.0, 100 mM EDTA, and 250 mM NaCl) and then vortexed thoroughly; 0.1 ml 10% SDS was added to the mixture and incubated at 37°C for 1 h. Then.15 ml 5 M NaCl and.13 ml 10 M NaCl + 10% CTAB were added to mixture, which was mixed gently and incubated at 65°C for 20 min. After cooling, 0.4 ml chloroform-isoamyl alcohol (24:1 v/v) was added to the mixture, which was thoroughly mixed and centrifuged for 15 min at 10,391 × *g*. The supernatants were pipetted into new 2-ml Eppendorf tubes containing 2 volumes of chilled 100% ethanol and kept under −20°C overnight to precipitate the DNA. The contents were centrifuged for 15 min at 10,391 × *g*, and the resulting supernatants were discarded. The precipitated DNAs were air-dried in a laminar air-flow chamber for 5–10 min. The dried DNAs were dissolved with 0.5 ml TE buffer containing 2 μl RNase and incubated for 1 h at 37°C. An equal volume of chloroform:isoamyl alcohol solution (24:1) was later added, mixed thoroughly, and centrifuged for 15 min at 10,391 × *g*. The supernatants were transferred into new 2-ml EP tubes. A twofold volume of chilled ethanol was added and incubated for about 2 h at −20°C. The contents were centrifuged at 10,391 × *g* for 15 min, and the resulting supernatants were discarded. The precipitated DNA pellets were washed with 70% ethanol and air-dried. The dried DNA pellets were re-suspended in 100 μl TE buffer and used as templates for PCR amplifications and other experiments.

### Total RNA Extraction and RT-PCR Assay

The strains were cultured in liquid CM for 4 days at a speed of 110 rpm. The mycelia were filtered, washed with sterilized ddH_2_O, dried with absorbent paper, and further dry frozen in liquid nitrogen. Total RNA was extracted from the individual strains using a HiPure Universal RNA kit (R4130-02; Magen, China). The expression of *MoOeIF3K* under different stress conditions and expression of individual subunits of the MoeIF3 complex in Δ*MoOeif3k* were monitored by quantitative real-time PCR (qRT-PCR) assays. Reverse transcription of RNAs was performed using the PrimeScript RT regent Kit with gDNA Eraser (RR047A; Takara, Japan). A 10-μl reaction mix was formulated as follows: 5 μl TB green, 3.4 μl RNase free water, 0.3 μl of each 10 μM forward and reverse primers listed in Supplementary Table 1 and 1 μl cDNA template. qRT-PCR was carried out with Eppendorf Realplex2 master cycler (AG 223341; Eppendorf, Hamburg, Germany). The raw qRT-PCR data were analyzed using the formula delta delta-CT (2^–ΔΔ*CT*^) method described by Rao et al. (2013) and Abdul et al. (2018). The expression level of tubulin was used as the reference or internal control. Error bars represent mean ± SD. The data were obtained from three independent biological experiments with three technical replicates for each independent experiment.

### Western-Blotting Analysis

The *MoOeIF3k*-GFP strain was cultured in a CM liquid medium for 4 days at a speed of 110 rpm. The mycelia were filtered and ground. About equal quantities (weight) of the ground samples were shared to a new CM liquid medium supplemented with different types of stress-inducing agent and MM medium, and incubated for 2 h at a speed of 110 rpm. Filtered individual samples were put into liquid nitrogen after drying out. The samples were ground into fine powder in liquid nitrogen, re-suspended in 5 ml protein lysis buffer (10 mM Tris–HCl, pH7.5, 150 mM NaCl, 0.5 mM EDTA, 0.5% NP40 for 1 L), well-mixed, and kept on ice for 30 min. The contents were centrifuged at 12,074 × *g* for 15 min at 4°C to remove cell debris. A protein analysis was performed using 12% SDS-PAGE gel and the samples were transferred to polyvinylidene fluoride (PVDF) membranes for Western blot assay. Anti-GFP and anti-actin were purchased from Abmart (United States) and Zoonbio (China), respectively, and used to detect total proteins in cell lysates from the individual strains.

For the Western blotting assays performed to monitor the expression of *MoOeIF3k* in response to the different types of stress-inducing osmolytes, the *MoOeIF3k* protein extracted from *MoOeIF3k*-GFP strains cultured on CM was used as normalization control (NC) for DTT, CR, and MM treatment while the expression level of *MoOeIF3k* protein recorded for *MoOeIF3k*-GFP strains pretreated with DMSO was used as the NC for Tu and Tg treatments. Actin-antibody was used as as the Western blot loading control, anti-beta Actin mAb from Zoonbio Biotechnology Co., Ltd., China catalog no. TE0303.

### Conidiation, Appressoria Formation, Turgor Pressure, Conidiophore Development, and Mating Assay

Conidia were washed from 10 day-old culture plates with sterilized ddH_2_O and filtered through three layers of lens paper into a 2-mL EP tube. For appressorium formation bioassays, 20 μl spore suspensions (concentration of 5 × 10^4^ spores per ml) were placed as droplets on hydrophobic coverslips and incubated under humid and dark conditions with a temperature of 28°C for 2, 4, 8, 16, and 24 h. More than 100 conidia were examined for each strain per experiment, with consistent results obtained from at least three independent biological experiments with three technical replicates. The turgor pressure level in the appressorium produced by conidia obtained from the individual strains on hydrophobic coverslips were assayed by treating appressorium with 1, 2, 3, and 4 M glycerin solution for 5 min. The rate of appressorium collapse was observed under a light microscope and used to measure appressorium integrity or turgor. At least 100 conidia/appressoria were observed per replicate; in all, a total of three biological experiments, with each consisting of three technical replicates, were carried out in this study.

Conidiophore staining assay was performed by cutting blocks of RBA media fully colonized by the individual strains. The cut blocks were placed on micro-slides with the side bearing the hyphae made to rest on the slide surface and incubated under light for 48 h at 28°C. After this period, the blocks were removed and stained with lactophenol cotton blue (100 ml LCB) solution (LCB solution; 20 ml phenol, 0.6 g cotton blue, 44 ml glycerine, and 16 ml lactic acid; distilled water was added to attain a final volume of 100 ml; the solution was diluted threefold before usage) for 5 min. Finally, the slides were washed with distilled water and visualized under an Olympus Bx51 microscope (Japan) using the bright field mode.

The mating assay was performed with the standard tester strain KA3 (*MAT1-1*, gifted by Dr. Didier Tharreau) with Δ*MoOeif3k* and Guy11 (*MAT1-2*) apart on an oatmeal agar (OA) medium at 20°C for 3–4 weeks.

### Pathogenicity Assay

For hyphae-mediated infection experiments, the individual strains were pre-cultured in liquid CM for 3 days in a shaking incubator at a speed of 120 rpm and a stable temperature of 28°C. The mycelia were filtered out and washed with sterilized double-distilled water. The excess water was drained off. The media-free mycelia were used as propagules to inoculate intact and injured barley leaves. The inoculated plants were first incubated in a dark chamber with stable relative humidity of 90% and a stable temperature of 25°C for 24 h. After 24-hpi, the inoculated tissues were transferred into a growth chamber with a photoperiod of 12-h light/12-h dark.

Under conidia mediated infection assays, spore suspensions were prepared with conidia obtained from the individual strains in a concentration of about 2–5 × 10^4^ per ml fortified with.02% v/v of Tween20 (addition of Tween20 helped in adhering the spores to the leaf tissues). The conidia suspensions were used to spray-inoculate 3 weeks-old blast-susceptible rice seedlings (O. *sativa* cv. CO39). The inoculated seedlings were incubated under the conditions described above for hyphae-mediated infection, under a dark and humid condition at 25°C for 24 h and transferred into the growth chamber with a 12-h light/12-h dark cycle. In both hyphae- and conidia-mediated infection assays, disease development and lesion severity were assessed 7 days post inoculation (dpi) and used as a measure of pathogenicity and virulence characteristics of the individual strains. Histopathological examinations (host penetration and colonization assays) were performed by inoculating the underside of the barley leaves with conidia suspension with a concentration of 2–5 × 10^4^ per ml. The inoculated tissues were incubated under the set of conditions stated above. The host invasion and colonization efficiencies of the individual strains were observed at 24-hpi by scanning the epidermal tissues of the inoculated barley leaves under a light microscope.

### Cytological Analysis

For glycogen staining, the appressoria were stained with a solution prepared from 60 mg/ml of KI and 10 mg/ml of I_2_ in distilled water (Thines et al., 2000; Xu et al., 2011; Zheng W. et al., 2015). Yellowish-brown glycogen deposits were visible immediately in the bright field. The samples were stained with Bodipy (D3922; Invitrogen, United States) containing10 μg/ml in phosphate-buffered saline (PBS) (Baerga et al., 2009; Zheng W. et al., 2015) during the comparative evaluation of lipid bodies.

### Microscopy Examinations

For microscopy, an Olympus DP72 fluorescent microscope or the Nikon A1 Plus confocal microscope (Japan) was used to observe the fluorescent light of GFP and mCherry. The emission and excitation wavelengths were 488 and 561 nm, respectively.

### Co-localization Assay

To confirm the localization of *MoOeIF3k*, we constructed the *MoOeIF3k*-GFP vector and co-transformed *MoOeIF3k*-GFP with His-mCherry and Kar2-mCherry markers into Guy11. The transformants were screened by PCR using the primer pair (eIF3K-OF and GFP-R) before using the microscope to screen the transformation strains. The His-mCherry (Zhang et al., 2019) and ER markers (Kar2-mCherry) (Zheng H. et al., 2015) were obtained from Dr. Lianhu Zhang at Jiangxi Agricultural University and Dr. Huawei Zheng at the Minjiang University.

### Co-immunoprecipitation Assay

For the immunoprecipitation of GFP fusion proteins from cellular extracts, the total proteins of *MoOeIF3k*-GFP and GFP strains were extracted and incubated with 30 μl of anti-GFP magarose beads (Smart-Life Sciences, China) for 4 h at 4°C. We then used a magnetic frame to wash the beads three times with 500 μl cold wash buffer (50 mM Tris, 0.15 M NaCl, and pH 7.4) and resuspended the anti-GFP magarose beads in 80 μl SDS-loading buffer. Proteins eluted from the anti-GFP magarose beads were analyzed by immunoblot detection with the anti-GFP antibodies (Abmart, China), followed by mass spectrometry (BGI, China).

### Quantification of Total Protein Levels Between the Individual Strains

Total proteins extracted from the mutant and wild-type strains were quantified using a BCA protein assay kit (BL521A; Biosharp, China). For a BCA working solution, BCA reagents labeled A and B were mixed in the ratio of 50:1.

Next, 20 μl of protein extracted from the individual strains were pipetted into 96-well microtiter plates (optimization was performed according to the instructions of the manufacturer), and 200 μl of the BCA working solution was added to the protein samples, mixed thoroughly with a micropipette, and kept under 37°C for 30 min.

Quantification of total protein content in the samples was recorded by taking readings at an absorbance of 562 nm using a multifunctional microplate reader (LB942; Berthold, China).

### Yeast Two-Hybrid Assay

To generate clone vectors and positive transgenic yeast strains for Y2H screening of probable MoeIF3k and subunits of the MoOCsn complex, the full-length cDNA of *MoOeIF3k* was amplified and cloned into a pGBKT7 plasmid containing the GAL4 DBD to obtain the bait vector *MoOeIF3k*-BD. The full-length cDNAs of MoOCsn1, MoOCsn2, MoOCsn3, MoOCsn4, MoOCsn5, MoOCsn6, MoOCsn7, and MoOCsn12 were amplified and cloned into pGADT7 plasmid harboring the Gal4 activation domain (AD) to obtain the individual prey vectors MoOCsn1-AD, MoOCsn2-AD, MoOCsn3-AD, MoOCsn4-AD, MoOCsn5-AD, MoOCsn6-AD, MoOCsn7-AD, and MoOCsn12-AD according to the protocol described by Young (1998). The interaction of pGBKT7-53 and pGADT7-T was used as the positive control, and pGBKT7-Lam and pGADT7-T were used as negative controls. The resultant bait and prey vectors were confirmed by sequencing and were co-transformed into the yeast strain AH109. All transformants were assayed with 1 × 10^6^ cells/μl droplet on SD-Leu-Trp and SD-Leu-Trp-His-Ade with 20 mg/ml X-α-gal plates.

### Bimolecular Fluorescence Complementation-Mediated *in vivo* Interaction Assays

The yellow fluorescence protein (YFP) was split into two halves fused to MoeIF3k and MoOCsn6 to obtain the *MoOeIF3k*-NYFP and MoOCsn5-CYFP constructs according to methods described by Zheng et al. (2018). The BiFC constructs were transformed into wild-type protoplasts according to the following combinations: MoOCsn5-CYFP + *MoOeIF3k*-NYFP, MoOCsn5-CYFP + NYFP, and CYFP + *MoOeIF3k*-NYFP. The transformants were screened on dual selection culture media containing hygromycin and neomycin. Potential candidates were isolated and confirmed by PCR. A microscopy examination of YFP fluorescence signals in the positive candidates harboring the pair of the split construct was performed with the Nikon A1 laser confocal microscope.

## Data Availability Statement

The datasets presented in this study can be found in online repositories. The names of the repository/repositories and accession number(s) can be found in the article/Supplementary Material.

## Author Contributions

LL, JN, and ZW conceived the study, designed the experiments, and wrote the manuscript. LL, JC, AD, QA, XC, SY, WB, and DZ conducted phenotype analysis and microscopy examination. All authors contributed to the article and approved the submitted version.

## Conflict of Interest

The authors declare that the research was conducted in the absence of any commercial or financial relationships that could be construed as a potential conflict of interest.

## Publisher’s Note

All claims expressed in this article are solely those of the authors and do not necessarily represent those of their affiliated organizations, or those of the publisher, the editors and the reviewers. Any product that may be evaluated in this article, or claim that may be made by its manufacturer, is not guaranteed or endorsed by the publisher.
