# Corrigendum: eIF3k Domain-Containing Protein Regulates Conidiogenesis, Appressorium Turgor, Virulence, Stress Tolerance, and Physiological and Pathogenic Development of *Magnaporthe oryzae Oryzae*

**DOI:** 10.3389/fpls.2021.807845

**Published:** 2021-11-24

**Authors:** Lili Lin, Jiaying Cao, Anqiang Du, Qiuli An, Xiaomin Chen, Shuangshuang Yuan, Wajjiha Batool, Ammarah Shabbir, Dongmei Zhang, Zonghua Wang, Justice Norvienyeku

**Affiliations:** ^1^State Key Laboratory of Ecological Pest Control for Fujian and Taiwan Crops, Fujian Agriculture and Forestry University, Fuzhou, China; ^2^Fujian University Key Laboratory for Plant-Microbe Interaction, Fujian Agriculture and Forestry University, Fuzhou, China; ^3^Key Laboratory of Green Prevention and Control of Tropical Plant Diseases and Pests, Ministry of Education, College of Plant Protection, Hainan University, Haikou, China; ^4^Institute of Oceanography, Minjiang University, Fuzhou, China

**Keywords:** *Magnaporthe oryzae Oryzae*, ribosomal RNA (rRNA), non-essential eIF3 complex, appressorium, nutrition starvation

In the article, as published, Zonghua Wang should also be listed as a corresponding author. The email address for the corresponding author Zonghua Wang is wangzh@fafu.edu.cn.

The authors apologize for this error and state that this does not change the scientific conclusions of the article in any way. The original article has been updated.

## Publisher's Note

All claims expressed in this article are solely those of the authors and do not necessarily represent those of their affiliated organizations, or those of the publisher, the editors and the reviewers. Any product that may be evaluated in this article, or claim that may be made by its manufacturer, is not guaranteed or endorsed by the publisher.

