# Supplementary material for: eIF3k Domain-Containing Protein Regulates Conidiogenesis, Appressorium Turgor, Virulence, Stress Tolerance, and Physiological and Pathogenic Development of Magnaporthe oryzae Oryzae
Source: Front Plant Sci. 2021 Oct 18;12:748120. doi: 10.3389/fpls.2021.748120 (PMC8558559; doi:10.3389/fpls.2021.748120)
Supplement: Supplementary file 1 [file Presentation_1.pdf]

eIF3k domain-containing protein regulates conidiogenesis, appressorium turgor, virulence, stress tolerance, physiological and pathogenic development of *Magnaporthe oryzae Oryzae*

Lili Lin[a], Jiaying Cao[a], Anqiang Du[a], Qiuli An[a], Xiaomin Chen[a], Shuangshuang Yuan[a], Wajjiha Batool[a], Ammarah Shabbir[a], Dongmei Zhang[a], Zonghua Wang[a, c], Justice Norvienyeku[a,b*]

a. State Key Laboratory of Ecological Pest Control for Fujian and Taiwan Crops & Fujian Universities Key Laboratory for Plant-microbe Interaction, Fujian Agriculture and Forestry University, Fuzhou, 350002, China.

b. Key Laboratory of Green Prevention and Control of Tropical Plant Diseases and Pests, Ministry of Education, College of Plant Protection, Hainan University, Haikou, 570228, China,

c. Institute of Oceanography, Minjiang University, Fuzhou, 350108, China.

d. *To whom correspondence should be addressed.Email: jk_norvienyeku@hainanu.edu.cn

List of supplementary tables and figures

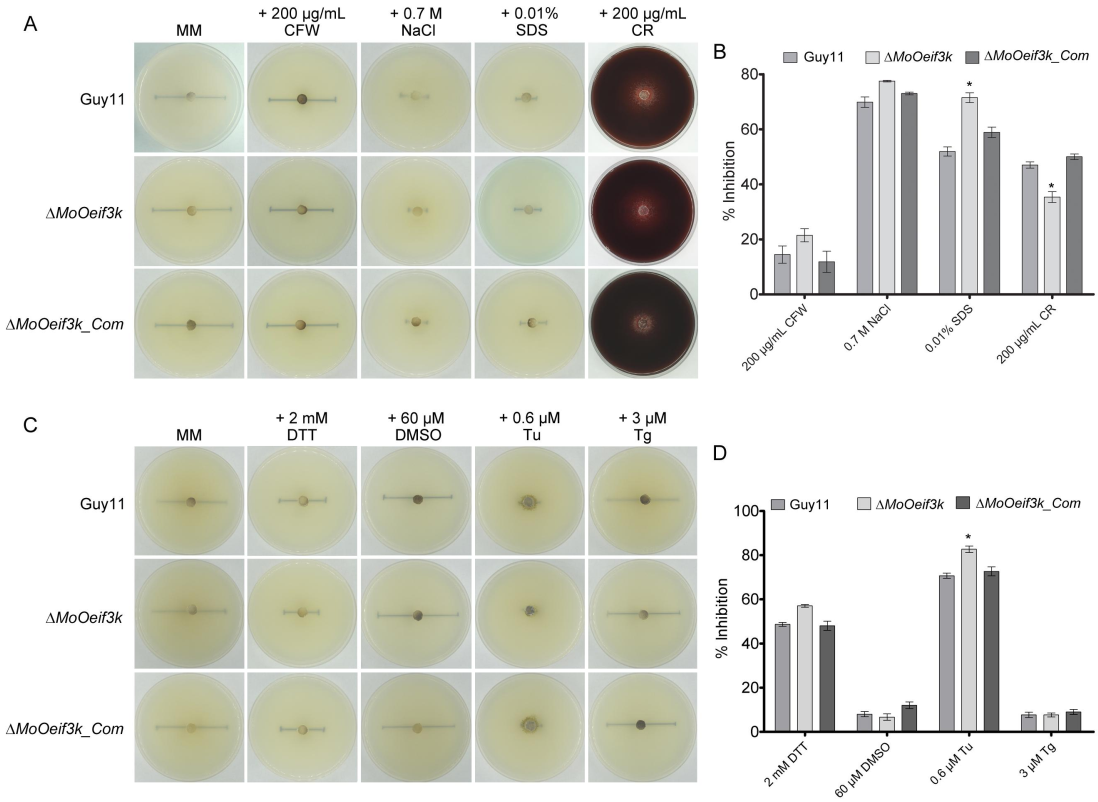

Supplementary figure S1: Δ*MoOeif3k* strains displayed differential response to multiple ER-stress inducing agents. (A) Growth response of the Δ*MoOeif3k* strains, the complemented strains, and wild-type cultured on MM supplemented with either 200 µg /mL Calcofluor White (CFW), 2.0 mM NaCl, 0.01% SDS or 200 µg/mL Conge Red (C.R.) as oxidative and ionic stress-inducing osmolytes. **(B)** A statistical representation of the inhibitory effects of oxidative and ionic stress-inducing osmolytes on the vegetative development of the Δ*MoOeif3k* strains, the complemented strains, and the wild-type strain. **(C)** Portrays the vegetative growth of the Δ*MoOeif3k*, the Δ*MoOeif3k_Com*, and wild-type strains MM supplemented with 2 mM DTT (Dithiothreitol), 60 µM DMSO (Dimethyl sulfoxide), 0.6 µM Tu (Tunicamycin), and 3.0 µM Tg (Thapsigargin) independently as ER-stress inducing agents. **(D)** A statistical representation of the inhibitory effects of different ER-stress inducing agents on the vegetative growth of the Δ*Moeif3k*, the Δ*Moeif3k_Com*, and wild-type

strains. **Note:** The inhibition data were generated from three independent biological experiments with five technical replicates each time. One-way ANOVA (non-parametric) statistical analysis was carried out with GraphPad-prism6, and Microsoft Excel spreadsheets and error bars represent the standard deviation. Inhibition rate = (the diameter of untreated strain – the diameter of treated strain)/ (the diameter of untreated strain) x100%. Single and double asterisks represent significant differences ($p \leq 0.05$ and $p \leq 0.02$), respectively.

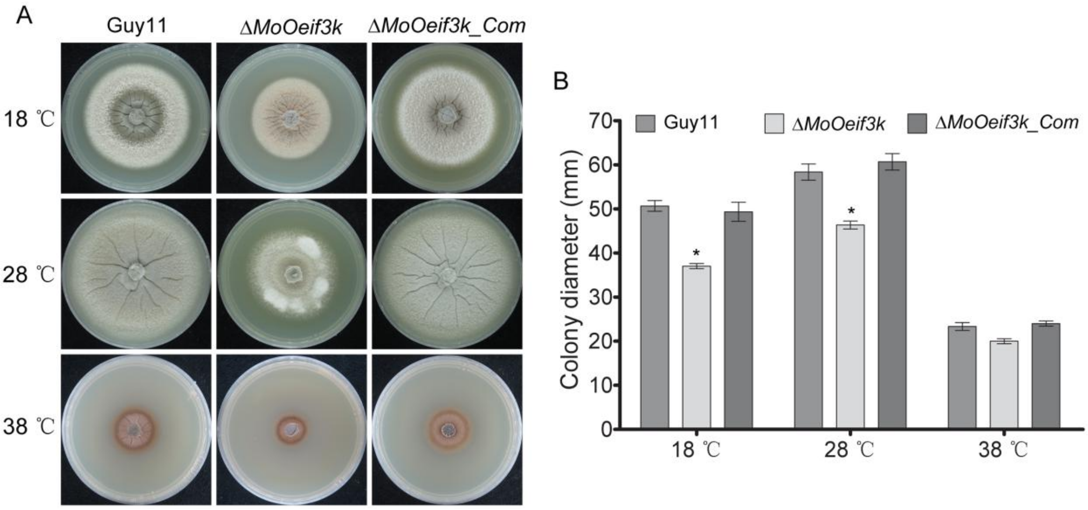

Supplementary figure S2: The response of Δ*MoOeif3k* strains to temperature variations. (A) Showed comparative growth performance of the Δ*MoOeif3k*, the Δ*MoOeif3k_Com* and the wild-type strain under temperatures that are 10˚C below or above the optimum growth temperature of 28˚C. **(B)** Statistical demonstration of average growth characteristics of the individual strains under different incubation temperatures. The data used for statistical computation were obtained from three independent biological experiments with three replicates each time with consistent results. One-way ANOVA (non-parametric) statistical analysis was carried-out with Graphpad-prism6 and Microsoft Excel spreadsheet. Error bars represent the standard deviation while asterisk "*" ($P \leq 0.05$) represent a

significant difference between the growth of the wild-type strain, the *ΔMoeif3k* strain, and the *ΔMoeif3k_Com* strains.

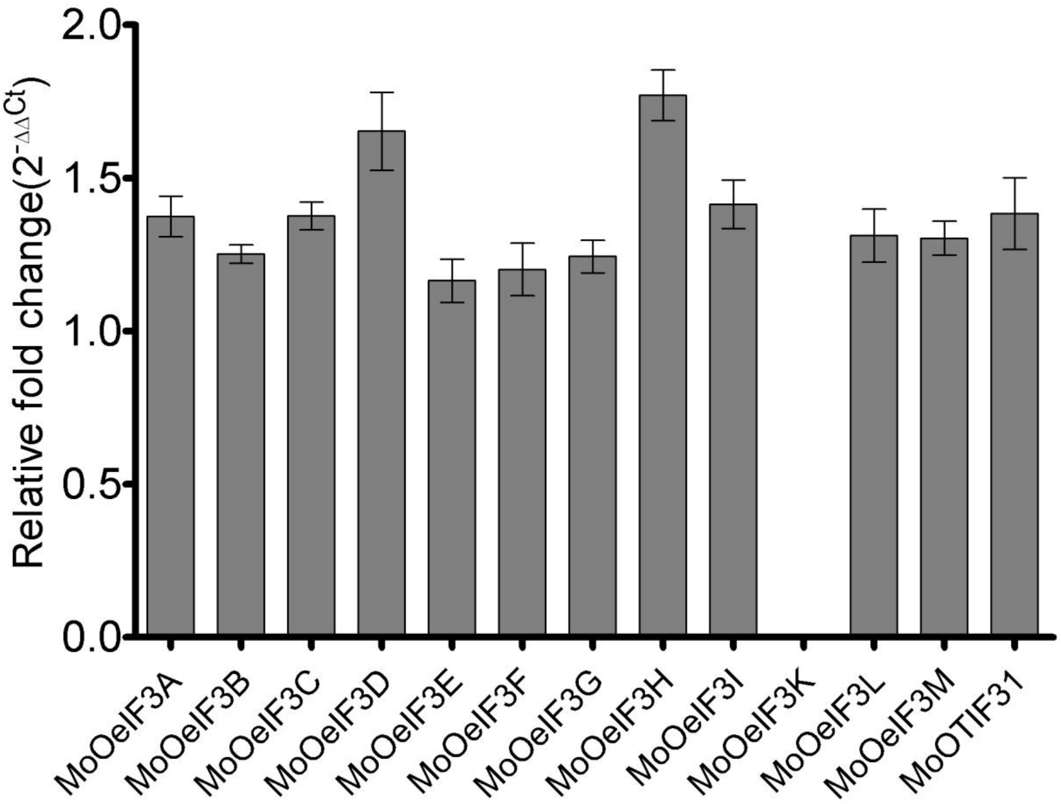

Supplementary figure S3: The impact of *MoOeIF3K* gene deletion on other putative eIF3 complex subunits' expression pattern. The Real-time quantitative PCR (qRT-PCR) data were analyzed with Microsoft excel spread and GraphPad prism6. Error bars represent mean ± SD. The analyses were carried out with data obtained for three biological experiments with three technical replications each time.

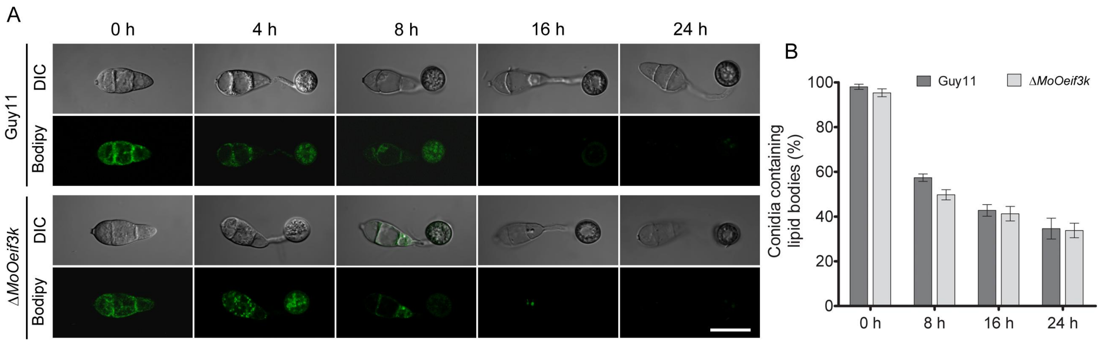

Supplementary figure S4: *MoOeIF3K* is dispensable in the mobilization, conidial-appressorium transport, and degradation of lipid bodies during pathogenic development of *MoO*. (A) Showed the dynamic mobilization and degradation lipids bodies in the wild-type strain, and the Δ*MoOeif3k* strains at different time points in appressorium development; Conidia obtained from the individual strains were incubated on hydrophobic coverslips and incubated. Lipid body dynamics during appressorium morphogenesis in the respective strains were observed under a confocal microscope after staining Bodipy at 0, 4, 8, 16, and 24 h post-incubation. Scale bar = 10μm. **(B)** A comparative statistical presentation of lipid body dynamics during pathogenic development of the wild-type strain, and the Δ*Moeif3k* strains. The data used for the statistical computation were obtained from three independent biological experiments with three replicates each time with consistent output. A total of 100 conidia or germinating/conidia with appressorium were counted at each time point in one biological experiment. Therefore total sample size （n） in three biological experiments per time point (n=100*3). Error bars represent the standard deviation.

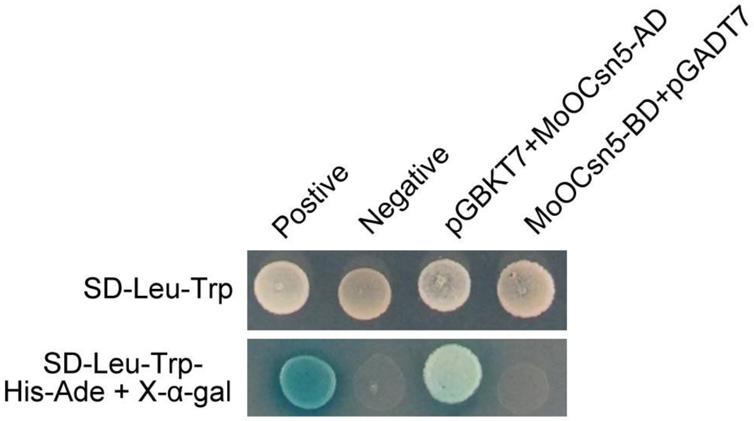

Supplementary figure S5: Showed the yeast strains harboring the empty pGBKT7 vector containing the GAL4 DB domain (vehicle) vs. MoOCsn5-AD and yeast strains harboring pGBKT7 vector containing the GAL4 AD domain (vehicle) vs. MoOCsn5-BD on SD, pGBKT7 vector containing the GAL4 DB domain (vehicle) vs. MoOCsn5-AD.

Table S1: List of premer pairs

Name	Primer	Primer binding site
eIF3K-OF	GACCGACTTGCGTTTGC	Position 321 from the N-terminal
eIF3K-OR	CCAGCCACTGCTCCATT	Position 199 from the C-terminal
eIF3K-AF	GAACAAAAGCTGGGTGGATGGCGTAAGAGGCA	Position 997 from the start codon of open reading frame
eIF3K-AR	CAGCGGCGCGCCGAATGTCGGGTGGAGGGATG	Position 54 from the start codon of open reading frame
eIF3K-BF	ACCGGGCCGGCCGGATTATGCGATCTTGGTGCTA	Position 139 from the stop codon of open reading frame

eIF3K-BR	GGTGGCGGCCGCTCTACTCCCAGACCGTTTCC	Position 1343 from the stop codon of open reading frame
eIF3K-UF	CTAGTAGGGACAAGAAATGGT	Position 1810 from the start codon of open reading frame
eIF3K-UR	GCAAAGTGCCGATAAACA	Position 203 from the stop codon of hygromycin sequence
YG/F	GATGTAGGAGGGCGTGGATATGTCCT	Position 117 from the start codon of hygromycin sequence
HY/RF	GTATTGACCGATTCCTTGCGGTCCGAA	Position 621 from the stop codon of hygromycin sequence
eIF3K-GFPF	GAACAAAAGCTGGGTGCTCGTCCGTGATGCCAAT C	Position 2051 from the start codon of open reading frame
eIF3K-GFPR	CTGCAGGCATGCAAGTGCAACCTCTTCCCATGCC C	The last 20 bp of the sequence of open reading frame without stop codon
GFP-R	GTTCACCTTGATGCCGTTC	Position 476 from the start codon of GFP sequence
eIF3K-qpcr-F	CGCTTCTGGGCCACTATC	Position 163 from the start codon of cDNA sequence
eIF3K-qpcr-R	TTCATCACGGAGGCTTGC	Position 420 from the stop codon of cDNA sequence
eIF3A-qpcr-F	ACAGGGTCCCATCTTCAA	Position 2827 from the start codon of cDNA sequence
eIF3A-qpcr-R	CAGCACGAGCAAGGTTAA	Position 25 from the stop codon of cDNA sequence
eIF3B-qpcr-F	GAAGTCTCAGGCAACAAC	Position 1301 from the start codon of cDNA sequence
eIF3B-qpcr-R	TCGTAGGTGCTTGAAGTT	Position 714 from the stop codon of cDNA sequence
eIF3C-qpcr-F	TACCACGAGAGGCAAATC	Position 1962 from the start codon of cDNA sequence
eIF3C-qpcr-R	GCGTCTCAATGCTCAATG	Position 404 from the stop codon of cDNA sequence
eIF3D-qpcr-F	TAAGGCAGATGTCCTCAAG	Position 1412 from the start codon of cDNA sequence
eIF3D-qpcr-R	CGCTTCAGACACATATCG	Position 151 from the stop codon of cDNA sequence
eIF3E-qpcr-F	TCGCATCTGAGATTCTGT	Position 574 from the start codon of cDNA sequence
eIF3E-qpcr-R	GAGGTCTGGATAGTGTTGA	Position 560 from the stop codon of cDNA sequence
eIF3F-qpcr-F	TGTCAATACTAGACCACGC	Position 122 from the start codon of cDNA sequence
eIF3F-qpcr-R	TCATGGCTGGTGGTGTAC	Position 766 from the stop codon

		of cDNA sequence
eIF3G-qpcr-F	GAACTCATCTCCGTCAGAA	Position 14 from the start codon of cDNA sequence
eIF3G-qpcr-R	CGTCGTCTTGACCTTTTG	Position 765 from the stop codon of cDNA sequence
eIF3H-qpcr-F	AACCACCATTCCTACATCTC	Position 650 from the start codon of cDNA sequence
eIF3H-qpcr-R	TTGCCACGCTGTAATCTT	Position240 from the stop codon of cDNA sequence
eIF3I-qpcr-F	GAGGCTAGGTTCTACCATAA	Position 804 from the start codon of cDNA sequence
eIF3I-qpcr-R	CTGCTCCATCTTGTTCTTC	Position 31 from the stop codon of cDNA sequence
eIF3L-qpcr-F	GCACTTCACAACCTACTACTA	Position 695 from the start codon of cDNA sequence
eIF3L-qpcr-R	CACCGAACTTCTCACGAA	Position 491 from the stop codon of cDNA sequence
eIF3M-qpcr-F	AGGAGAACCTTGACAATGA	Position 838 from the start codon of cDNA sequence
eIF3M-qpcr-R	CCGAGTGAATCAGGAAGA	Position 257 from the start codon of cDNA sequence
TIF31-qpcr-F	ATCACGACCATCAACAAC	Position 3351 from the start codon of cDNA sequence
TIF31-qpcr-R	CTCTCCTTCATCTTGACAAC	Position 399 from the start codon of cDNA sequence
MoeIF3k-BDF	CTGATCTCAGAGGAGGACCTGCATATGCCTCGTCTTCCCAACAG	The first 20 bp of the cDNA sequence
MoeIF3k-BDR	CGCTGCAGGTCGACGGATCCCCGGGAATCATGCAACCTCTTCCCATG	The last 20 bp of the cDNA sequence
MoCsn1-ADF	GACGTACCAGATTACGCTCATATGGCGACTCCTCATGAGAA	The first 20 bp of the cDNA sequence
MoCsn1-ADR	TATCGATGCCCACCCGGGTGGAATCAAAATACGGATGCCATCG	The last 20 bp of the cDNA sequence
MoCsn2-ADF	GACGTACCAGATTACGCTCATATGTCCGACGACGACTTCAT	The first 20 bp of the cDNA sequence
MoCsn2-ADR	TATCGATGCCCACCCGGGTGGAATCATGTAAAGGTCGAATGAACTG	The last 23 bp of the cDNA sequence
MoCsn3-ADF	GACGTACCAGATTACGCTCATATGGATCACTGCGCGTC	The first 17 bp of the cDNA sequence
MoCsn3-ADR	TATCGATGCCCACCCGGGTGGAACTACTGCCCAGATACTATGCC	The last 21 bp of the cDNA sequence
MoCsn4-ADF	GACGTACCAGATTACGCTCATATGGCCTCCGACTCGATAAA	The first 20 bp of the cDNA sequence

MoCsn4-ADR	TATCGATGCCCACCCGGGTGGAATCAGACTACCAAGTTGGCCG	The last 20 bp of the cDNA sequence
MoCsn5-ADF	GACGTACCAGATTACGCTCATATGGATGTTGCTATGAAGTCG	The first 21 bp of the cDNA sequence
MoCsn5-ADR	TATCGATGCCCACCCGGGTGGAACTACGACGCAGCCGACG	The last 17 bp of the cDNA sequence
MoCsn6-ADF	GACGTACCAGATTACGCTCATATGGCTTCAGAAATGGAGAC	The first 21 bp of the cDNA sequence
MoCsn6-ADR	TATCGATGCCCACCCGGGTGGAATCACGACATGATATCGCC	The last 17 bp of the cDNA sequence
MoCsn7-ADF	GACGTACCAGATTACGCTCATATGGAGCAAGCAAAGGCT	The first 20 bp of the cDNA sequence
MoCsn7-ADR	TATCGATGCCCACCCGGGTGGAATCAAAGCTTTCTTCGACTCG	The last 19 bp of the cDNA sequence
MoCsn12-ADF	GACGTACCAGATTACGCTCATATGGACAAACTCTTCGATCA	The first 21 bp of the cDNA sequence
MoCsn12-ADR	TATCGATGCCCACCCGGGTGGAATCAGATATTAGTACCAGGAAATG	The last 23 bp of the cDNA sequence

Supplementary Table S2: List and quantitative attributes of Transcription factors recovered exclusively from MoeIF3k-GFP immuno-complex.

Gene ID	Annotation	Domain	Protein_Qscore
MGG_00692	cell pattern formation-associated protein stuA	KilA-N domain	2.476466975
MGG_06848	hypothetical protein	Zinc finger, C2H2 type	4.990595851
MGG_05033	Fungal-specific Zn(2)-Cys(6) domain-containing transcription factor, putative	Fungal Zn(2)-Cys(6) binuclear cluster domain	4.990595851
MGG_04674	Fungal-specific Zn(2)-Cys(6) domain-containing transcription factor, putative	Fungal Zn(2)-Cys(6) binuclear cluster domain	17.88923707
MGG_08168	hypothetical protein	Fungal Zn(2)-Cys(6) binuclear cluster domain	7.812670276
MGG_08829	transcriptional repressor rco-1	WD domain, G-beta repeat<br>Tup N-terminal	36.45606712
MGG_10575	hypothetical protein	Helix-loop-helix DNA-binding domain	7.923119954
MGG_17277	Histone chaperone Rttp106-like	histone chaperone RTT106	7.521622804
MGG_15156	phosphatidylinositol 3-kinase tor2	FAT domain<br>Phosphatidylinositol 3- and 4-kinase<br>Domain of unknown function (DUF3385)<br>FKBP12-rapamycin binding domain<br>FATC domain<br>HEAT repeat	9.115096497
MGG_02476	hypothetical protein	CCR4-Not complex	24.38491403

		component, Not1<br>CCR4-NOT transcription complex subunit 1 CAF1-binding domain<br>Domain of unknown function (DUF3819)<br>CCR4-NOT transcription complex subunit 1 TTP binding domain<br>CCR4-NOT transcription complex subunit 1 HEAT repeat	
MGG_02784	DNA-binding protein SMUBP-2	SEN1 N terminal<br>AAA domain	8.388495798
MGG_11764	Fungal-specific Zn(2)-Cys(6) domain-containing transcription factor, putative	Fungal Zn(2)-Cys(6) binuclear cluster domain<br>Fungal specific transcription factor domain	3.715062171
MGG_04878	Lid2 complex component lid2	PLU-1-like protein<br>JmjC domain, hydroxylase<br>ARID/BRIGHT DNA binding domain<br>PHD-finger<br>jmjN domain<br>C5HC2 zinc finger	4.717813221
MGG_04652	DNA-directed RNA polymerase II largest subunit	RNA polymerase Rpb1, domain 1<br>RNA polymerase Rpb1, domain 5<br>RNA polymerase Rpb1, domain 2<br>RNA polymerase Rpb1,	49.32142726

		domain 6<br><br>RNA polymerase Rpb1, domain 3<br><br>RNA polymerase Rpb1, domain 7<br><br>RNA polymerase Rpb1, domain 4	
MGG_03196	RCM-1	Tetratricopeptide repeat	4.990595851
MGG_07291	CMGC/CDK/CRK7 protein kinase	Protein kinase domain	4.990595851
MGG_06470	DNA repair helicase rad25	ERCC3/RAD25/XPB C-terminal helicase<br><br>Helicase conserved C-terminal domain<br><br>Type III restriction enzyme, res subunit	38.61131965
MGG_01012	ISWI chromatin-remodeling complex ATPase ISW2	SNF2 family N-terminal domain<br><br>SLIDE<br><br>HAND<br><br>Helicase conserved C-terminal domain	4.990595851
MGG_11779	hypothetical protein	AAA domain	55.47244606
MGG_01159	Histone H3	Core histone H2A/H2B/H3/H4	4.216782466
MGG_07316	FACT complex subunit spt-16	FACT complex subunit SPT16 N-terminal lobe domain<br><br>FACT complex subunit (SPT16/CDC68)<br><br>Metallopeptidase family M24<br><br>Histone chaperone	155.8731848

		Rttp106-like	
MGG_08015	hypothetical protein	Zinc finger, C2H2 type	4.990595851
MGG_02289	Fungal-specific Zn(2)-Cys(6) domain-containing transcription factor, putative	Fungal Zn(2)-Cys(6) binuclear cluster domain	2.004340727
MGG_05204	transcription initiation factor TFIID subunit 14	YEATS family<br>Bromodomain extra-terminal - transcription regulation	4.990595851
MGG_11724	hypothetical protein	Fungal Zn(2)-Cys(6) binuclear cluster domain	4.762874702
MGG_06321	glycyl-tRNA synthetase	Anticodon binding domain<br>tRNA synthetase class II core domain (G, H, P, S and T)	8.108156424
MGG_09562	hypothetical protein	Fungal specific transcription factor domain<br>Fungal Zn(2)-Cys(6) binuclear cluster domain	13.54211034
MGG_04477	DNA-directed RNA polymerase III subunit RPC1	RNA polymerase Rpb1, domain 1<br>RNA polymerase Rpb1, domain 5<br>RNA polymerase Rpb1, domain 2<br>RNA polymerase Rpb1, domain 3<br>RNA polymerase Rpb1, domain 4	2.419221079
MGG_06984	DNA-directed RNA polymerase III subunit RPC2	RNA polymerase Rpb2, domain 6<br>RNA polymerase beta subunit<br>RNA polymerase Rpb2, domain 7	8.448996764

		RNA polymerase Rpb2, domain 2<br>RNA polymerase Rpb2, domain 4<br>RNA polymerase Rpb2, domain 3<br>RNA polymerase Rpb2, domain 5	
MGG_02493	hypothetical protein	ARID/BRIGHT DNA binding domain	8.705658022
MGG_03215	DNA-directed RNA polymerase II subunit RPB3	RNA polymerase Rpb3/RpoA insert domain<br>RNA polymerase Rpb3/Rpb11 dimerisation domain	12.33769896
MGG_06381	hypothetical protein	A49-like RNA polymerase I associated factor	8.448996764
MGG_09284	DNA-directed RNA polymerase I and III subunit RPAC	RNA polymerase Rpb3/RpoA insert domain<br>RNA polymerase Rpb3/Rpb11 dimerisation domain	12.93254449
MGG_07728	hypothetical protein	DNA polymerase phi	40.87768826

Supplementary Table S3: Total interactors identified exclusively recovered from MoOeIF3k-GFP immuno-complex and their corresponding quantitative attributes.

Gene ID	Annotation	Protein_Qscore_average	coverage_average	Unique_Peptide_Num_average	Unique_Spectra_Num_average	Abundance_average	iBAQ_average
MGG_09480	hypotheticalprotein	10.44990312	0.186966667	3	3	2434812.573	202901.0478
MGG_17596	hypotheticalprotein	4.917030281	0.062733333	1	1	2744897.031	130709.3824
MGG_01606	methylmalonate-semialdehydedehydrogenase	4.0261661	0.050733333	1	1	27759197.99	750248.5944
MGG_11193	bystin	16.33295672	0.130566667	4	4	5487541.706	161398.2855
MGG_04201	U3smallnucleolarribonucleoproteinIMP3	15.17947886	0.240466667	3	5	50355225.83	3596801.845
MGG_09529	mitochondrial37SribosomalproteinSWS2	17.0295485	0.3165	4	5	7400707.117	740070.7117
MGG_02648	interferon-inducedGTP-bindingproteinMx	59.5893254	0.248266667	15	18	60875375.51	1352786.122
MGG_04007	hypotheticalprotein	37.44167212	0.225133333	9	10	26753349.27	891778.3091
MGG_08144	Ras-likeproteinRab7	9.107339908	0.152866667	2	3	6346942.141	396683.8838

MGG_04113	Ribose-5-phosphateisomerase	8.134034753	0.0488	2	2	2810663.912	140533.1956
MGG_07731	Cullin-3	24.92845888	0.087966667	6	8	7845075.701	156901.514
MGG_00491	superoxidedismutase	33.26628436	0.3963	7	12	57594303.65	4799525.304
MGG_10312	hypotheticalprotein	12.81684355	0.081033333	4	4	32725198.78	861189.4415
MGG_10677	mannose-6-phosphateisomerase	11.39603167	0.094266667	3	3	2722939.416	118388.6703
MGG_03838	Ser/Thrproteinphosphatase	17.91492162	0.069666667	4	4	3155049.861	83027.62791
MGG_05401	bifunctionalP-450:NADPH-P450reductase	8.458797232	0.024233333	2	2	1234578.782	18993.51972
MGG_05651	caseinkinaseIIsubunitbeta-2	13.91575353	0.177066667	3	4	7405248.042	617104.0035
MGG_04156	aspartateaminotransferase	8.894481221	0.0591	2	2	3313745.044	157797.3831
MGG_01364	hypotheticalprotein	4.340054411	0.0158	1	1	1092374.419	18834.0417
MGG_08880	hypotheticalprotein	20.29600189	0.1104	5	6	23412963.48	668941.8136
MGG_16895	Eukaryotictranslationinitiationfactor3subunitC	285.5538127	0.6358	63	224	3151614329	63032286.57
MGG_11764	Fungal-specificZn(2)-Cys(6)domain-containingtranscriptionfactor,putative	4.647170906	0.0151	1	1	450477.3238	22523.86619
MGG_01154	hypotheticalprotein	78.85433867	0.557333333	17	29	158115281.6	9882205.101

MGG_00291	ankyrinrepeatdomain-containingprotein28	5.072348799	0.0068	1	1	283507.0557	2922.753151
MGG_04008	60SribosomalproteinL4	17.45582202	0.155966667	5	6	10605695.94	461117.2149
MGG_03210	U3smallnucleolarRNAassociatedprotein	25.51871858	0.194	6	7	11003502.15	458479.2564
MGG_16213	hypotheticalprotein	13.30979349	0.027033333	3	3	5264632.897	61216.66159
MGG_00129	hypotheticalprotein	8.421783131	0.0636	2	2	2498357.8	131492.5158
MGG_05266	eukaryotictranslationinitiationfactor3	145.7628977	0.6837	31	122	1025743930	42739330.43
MGG_12141	hypotheticalprotein	5.877571341	0.0147	1	2	2604504.219	34269.79236
MGG_06914	GTP-bindingproteinrhb1	11.85297041	0.226966667	2	4	2635482.448	239589.3135
MGG_06298	hypotheticalprotein	3.447141144	0.044333333	1	1	2919313.23	162184.0684
MGG_05249	chaperonednaJ3	35.25572316	0.274666667	9	12	48892855	2037202.292
MGG_01806	hypotheticalprotein	3.139162797	0.031	1	1	772928.9517	30917.15807
MGG_05136	pre-rRNA-processingproteinPNO1	7.420049522	0.0947	2	2	6681031.687	513925.5144
MGG_01013	eukaryotictranslationinitiationfactor339kDasubunit	132.8065823	0.8358	29	105	965332418.8	40222184.12
MGG_06345	prolyl-tRNAsynthetase1	12.22905356	0.055733333	3	3	10551936.9	263798.4225

MGG_16457	hypotheticalprotein	9.904648945	0.047	3	3	2954861.328	51839.67242
MGG_03714	annexinANXC4	6.105736632	0.028966667	2	2	22334188.07	519399.7226
MGG_01802	chitinsynthase1	2.93056929	0.0086	1	1	140765.2703	3433.299277
MGG_01279	CAMK/CAMKL/KIN1proteinkinase	34.21728183	0.115166667	8	13	20013630.59	384877.5113
MGG_02390	hypotheticalprotein	11.50582503	0.0907	3	3	2146154.947	76648.39097
MGG_06135	GTP-bindingproteinypt2	4.812247143	0.1812	1	2	1646452.04	96850.11999
MGG_07030	hypotheticalprotein	25.63461505	0.1423	6	8	18662935.61	491129.8844
MGG_11241	generalstressresponseproteinWhi2	2.671855342	0.0207	1	1	158106.321	13175.52675
MGG_03597	hypotheticalprotein	55.01398876	0.226566667	13	17	37545843.1	915752.2707
MGG_01224	WDrepeat-containingprotein	46.43067569	0.155766667	11	15	53360257.01	1270482.31
MGG_02453	rRNAprocessingproteinEbp2	35.64112054	0.2735	8	17	46047925.06	2558218.059
MGG_01362	CMGC/CDK/CDC2proteinkinase	12.06702342	0.1146	3	4	13016745.28	565945.4467
MGG_05706	hypotheticalprotein	5.072348799	0.0245	1	2	220725.4434	4504.600885
MGG_00952	amidohydrolase2	8.196097931	0.0671	2	3	8278835.498	258713.6093

MGG_03269	hypotheticalprotein	6.651799869	0.0192	2	2	67093327.22	894577.6963
MGG_17001	actin-likeprotein2	3.944251431	0.030166667	1	1	825838.211	34409.92546
MGG_01116	hypotheticalprotein	14.50624692	0.281566667	4	4	6168640.199	474510.7846
MGG_05647	50SribosomalproteinL2	12.06601219	0.0842	4	4	18828247.87	784510.3278
MGG_15860	54SribosomalproteinL4	8.488655857	0.092733333	3	3	17702044.66	885102.2332
MGG_10655	delta-aminolevulinicaciddehydratase	4.647170906	0.0283	1	1	708239.6965	32192.71348
MGG_01846	AP-3complexbeta3Bsubunit	10.23495491	0.036533333	3	3	3216405.272	64328.10545
MGG_03202	replicationfactorCsubunit5	11.00195065	0.093766667	3	3	25358760.78	1102554.816
MGG_18017	hypotheticalprotein	6.676541474	0.057566667	2	2	1492268.706	74613.43531
MGG_11547	hypotheticalprotein	73.12740272	0.306666667	17	23	113887777	3349640.499
MGG_02896	hypotheticalprotein	26.08519735	0.277433333	6	8	41890041.68	2327224.538
MGG_03164	Cofilin	24.01738524	0.5263	5	8	32047839	3560871
MGG_06676	hypotheticalprotein	6.946888842	0.095966667	2	2	2389558.539	199129.8783
MGG_11336	hypotheticalprotein	6.333073287	0.070033333	1	1	5142920.932	367351.4951

MGG_02781	EFR3	22.08056374	0.0521	6	6	16887292.97	255868.0753
MGG_03855	hypotheticalprotein	100.0787084	0.8404	22	362	124745523.8	8910394.558
MGG_10382	ribosomebiogenesisproteinRPF2	63.37611757	0.572566667	15	30	108638145.5	6390479.147
MGG_00361	hypotheticalprotein	15.40078917	0.071933333	4	5	9829312.757	280837.5073
MGG_10835	ribosomebiogenesisproteinnsa-2	11.85888942	0.130766667	3	3	5914065.362	347886.1978
MGG_16920	hypotheticalprotein	10.24355776	0.172333333	3	4	4042503.396	404250.3396
MGG_04693	WDrepeat-containingprotein57	20.54825172	0.1979	6	7	10448109.57	549900.5036
MGG_03459	Atg26p	3.058944723	0.007466667	1	1	3699421.501	38138.36599
MGG_05957	50SribosomalproteinL1	12.4692183	0.1521	3	3	14247283.62	712364.181
MGG_08343	proteasomesubunitalphatype-4	12.97391973	0.1527	3	3	4001601.355	250100.0847
MGG_04993	ATP-dependentRNAhelicaseDBP9	13.07821972	0.0718	3	3	11854400.2	320389.1947
MGG_03271	hypotheticalprotein	27.43901166	0.1906	6	7	43067841.03	1485097.967
MGG_06329	S-(hydroxymethyl)glutathionedehydrogenase	5.072348799	0.0925	1	1	4134961.019	243233.0011
MGG_10196	bromodomaincontaining1	6.006586691	0.0151	2	2	112276.3807	1969.761065

MGG_11536	Alpha-xylosidase	3.310761773	0.015233333	1	1	1765032.497	36021.07136
MGG_06468	hypotheticalprotein	17.18688263	0.1746	5	5	9959443.743	663962.9162
MGG_04330	mitochondrialribosomalproteinsubunitS4	4.750178385	0.031366667	2	2	7188643.508	287545.7403
MGG_06678	ribose-phosphatepyrophosphokinase5	32.22535699	0.240266667	7	7	24955508.6	1085022.113
MGG_03196	RCM-1	5.072348799	0.015166667	1	1	2672079.499	78590.57351
MGG_00106	budsiteselectionprotein7	12.40176622	0.048433333	3	3	1788683.903	37264.24799
MGG_12738	Xanthinedehydrogenase	4.523592904	0.011333333	1	1	1378307.84	18377.43787
MGG_01987	C2H2fingerdomain-containingprotein	27.49363936	0.0848	6	7	21855086.61	496706.514
MGG_02927	hypotheticalprotein	9.482088477	0.0294	2	2	2485418.895	46894.69613
MGG_05949	acyl-CoAdehydrogenase	13.34172759	0.068866667	3	3	11730826.65	469233.0659
MGG_02943	hypotheticalprotein	5.072348799	0.0506	1	2	0	0
MGG_05810	ATP-dependentRNAhelicaseDBP7	8.639954257	0.035966667	2	2	4950323.325	103131.7359
MGG_05048	hypotheticalprotein	74.55312795	0.261	19	22	69848942.82	1396978.856
MGG_06124	activatingsignalcointegrator1complexsubunit3	38.93695365	0.062233333	10	10	46345926.67	429128.9506

MGG_00849	hypotheticalprotein	9.808727902	0.084066667	3	3	9655234.523	603452.1577
MGG_09493	AP-2complexsubunitalpha	9.549361658	0.027966667	2	2	11929031.22	205672.9521
MGG_07136	nucleolarGTP-bindingprotein1	53.89367813	0.258	13	16	36306353.04	907658.8261
MGG_08168	hypotheticalprotein	11.87996528	0.045566667	3	3	5093360.586	159167.5183
MGG_06242	ribosomebiogenesisproteinKri1	23.79143015	0.1092	5	6	18702367.25	550069.625
MGG_17936	hypotheticalprotein	8.734483152	0.066633333	2	2	12376129.45	651375.2342
MGG_09284	DNA-directedRNApolymeraseIandIIIsubunitRPAC1	14.87192333	0.122433333	3	3	3696594.408	205366.356
MGG_00457	hypotheticalprotein	9.180325102	0.067266667	2	3	2643401.639	155494.214
MGG_01524	60SribosomalproteinL3	20.68633059	0.188866667	5	5	10247163.57	465780.1625
MGG_03587	essentialformitoticgrowth1	26.2458388	0.311866667	7	9	32230641.09	1790591.172
MGG_05248	60SribosomalproteinL32	18.79279625	0.430033333	4	14	52863198.3	8810533.049
MGG_12920	hypotheticalprotein	7.352860124	0.067666667	2	2	3439283.527	143303.4803
MGG_10265	hypotheticalprotein	12.32577694	0.123866667	3	3	6285899.494	349216.6386
MGG_06397	DNApolymerasealphacatalyticsubunit	32.29407358	0.075833333	9	10	15281253.84	198457.8421

MGG_07271	mitochondrialribosomalsmallsubunitcomponent	58.41888006	0.383	14	16	105073326	4378055.25
MGG_07156	hypotheticalprotein	27.73851656	0.135	6	7	39052770.04	1084799.168
MGG_08053	WDrepeat-containingproteinJIP5	18.11404105	0.174566667	4	4	12202971.69	530563.9866
MGG_00172	glycoproteasepgp1	11.02281482	0.052566667	3	3	3137414.066	87150.39072
MGG_05648	hypotheticalprotein	27.09518317	0.336433333	6	8	15129470.64	1080676.474
MGG_17888	hypotheticalprotein	7.011582774	0.206466667	2	2	5614211.242	935701.8737
MGG_04966	lysyl-tRNAsynthetase	11.57605943	0.049333333	3	3	3736989.697	113242.112
MGG_01026	hydroxymethylglutaryl-CoAsynthase	7.964566646	0.083333333	2	3	4581276.366	218156.0174
MGG_07117	NADPH-cytochromeP450reductase,NADPH-cytochromeP450reductase,variant	13.73037222	0.070033333	4	4	35786731.01	832249.5583
MGG_01381	calciumpermease	4.010112142	0.0066	2	2	5494393.9	148497.1324
MGG_09834	catalase-peroxidase2	2.461902386	0.0076	1	1	811787.1583	20294.67896
MGG_00692	cellpatternformation-associatedproteinstuA	2.40445486	0.0129	1	1	450326.068	26489.76871
MGG_04012	3-hydroxyisobutyryl-CoAhydrolase	55.81563499	0.2819	12	16	127716098.5	4404003.398
MGG_07145	cullin-1	74.58126815	0.303333333	19	24	144559861.6	2891197.231

MGG_06404	hypotheticalprotein	9.873362076	0.098833333	3	3	13218005.96	574695.9114
MGG_10652	digestiveorganexpansionfactor	19.78908246	0.092333333	5	5	10327175.8	303740.4646
MGG_07449	hypotheticalprotein	11.11658418	0.151533333	3	3	6061413.833	505117.8194
MGG_04495	phosphoglucomutase	11.44350361	0.061966667	3	4	4688883.049	133968.0871
MGG_12603	hypotheticalprotein	9.128685932	0.136266667	2	2	4761387.489	264521.5272
MGG_10633	AP-1complexsubunitmu-1	36.30409295	0.270833333	8	10	28671613.1	988676.3137
MGG_01160	HistoneH4	25.47396356	0.485433333	6	9	46648938.97	7774823.162
MGG_07626	CytochromeP450monooxygenase	14.66283541	0.1074	5	5	12370109.17	374851.7931
MGG_07323	histoneacetyltransferasetypeBsubunit2	5.314150779	0.035166667	2	2	749841.4651	31243.39438
MGG_17277	histonechaperoneRTT106	8.552788953	0.039866667	2	2	2378281.14	95131.24558
MGG_00087	ribosomebiogenesisproteinSSF1	16.36963667	0.127966667	3	4	5665889.75	269804.2738
MGG_09458	GTPcyclohydrolase1	40.98841978	0.420466667	10	15	37063276.46	1853163.823
MGG_04746	DOM34-interactingprotein2	40.65287761	0.1171	12	12	30434359.21	515836.5969
MGG_16854	hypotheticalprotein	13.94469566	0.060866667	3	4	2341319.225	61613.66381

MGG_13527	hypotheticalprotein	5.80003083	0.018	1	1	943386.2675	21939.21552
MGG_10171	hypotheticalprotein	20.1193472	0.160433333	4	4	10636094.31	531804.7156
MGG_09926	Glucose-6-phosphate1-dehydrogenase	14.77188876	0.093366667	4	4	5546429.593	138660.7398
MGG_01509	glycinerichprotein	3.350749172	0.1653	1	1	0	0
MGG_10140	hypotheticalprotein	16.71983297	0.195866667	4	4	9101260.352	535368.256
MGG_06392	ornithineaminotransferase	7.770980848	0.0573	2	2	13760876.26	509662.0838
MGG_09372	hypotheticalprotein	36.78765285	0.2062	8	12	54630360.08	1951084.289
MGG_04878	Lid2complexcomponentlid2	6.346398251	0.0133	2	2	12362208.09	130128.5062
MGG_05282	50SribosomalproteinL13	9.342183223	0.1676	2	3	15572382.65	1297698.554
MGG_02504	elongationfactorTuGTPbindingdomain-containingprotein	13.16351153	0.084666667	3	4	5717138.08	184423.809
MGG_00341	eukaryotictranslationinitiationfactor3subunitD	134.1410019	0.6335	30	93	719740507.5	26657055.83
MGG_08924	hypotheticalprotein	32.70949015	0.278866667	7	11	23631431.92	1817802.455
MGG_07716	DnaJdomain-containingprotein	3.941370259	0.021	1	1	1386584.611	69329.23057
MGG_03120	30SribosomalproteinS6	21.06476579	0.356766667	5	5	14531819.04	1614646.56

MGG_00101	ATPaseNPA3	11.33671343	0.0621	2	3	2660855.922	147825.329
MGG_06483	ATP-dependentRNAhelicaseDBP8	9.405266407	0.0478	2	2	2911157.655	83175.93301
MGG_00567	hypotheticalprotein	81.4745461	0.1583	21	22	65042915.58	656999.1473
MGG_01978	eukaryotictranslationinitiationfactor3subunitB	250.5327453	0.7641	54	153	2055807960	54100209.48
MGG_10192	Eukaryotictranslationinitiationfactor3subunitA	278.3808289	0.6114	64	209	2734761976	41435787.51
MGG_17694	hypotheticalprotein	5.946304885	0.062166667	1	1	4258242.492	304160.178
MGG_03654	hypotheticalprotein	3.450095603	0.0546	1	1	739516.9518	49301.13012
MGG_09499	Ras-2	7.257066492	0.077433333	2	3	5823395.688	485282.974
MGG_17942	hypotheticalprotein	38.56013559	0.117633333	10	11	26117066.34	384074.5049
MGG_03086	U2snRNPcomponentIST3	9.729714252	0.0808	3	3	25966153	1854725.214
MGG_11229	hypotheticalprotein	9.83457879	0.036566667	2	2	4831551.194	155856.4901
MGG_02540	isovaleryl-CoAdehydrogenase2	5.105336723	0.041866667	2	2	4152032.92	173001.3717
MGG_15026	hypotheticalprotein	7.150186553	0.0445	3	3	20409827.31	551616.9543
MGG_10522	hypotheticalprotein	27.34680716	0.251666667	6	8	20616156.98	859006.5408

MGG_02665	hypotheticalprotein	2.761252159	0.0054	1	1	1948390.717	20950.43782
MGG_00851	hypotheticalprotein	3.664303097	0.0259	1	1	1322109.359	55087.88997
MGG_01501	hypotheticalprotein	9.08307884	0.089433333	2	2	5255786.584	250275.5516
MGG_02853	nucleasePA3	9.460441336	0.089333333	2	2	5441727.307	340107.9567
MGG_17452	hypotheticalprotein	10.99190764	0.0302	3	3	4057673.114	61479.89567
MGG_03116	spliceosomecomponent	11.94087509	0.0769	3	3	11905268.36	661403.7976
MGG_14679	tryptophanyl-tRNAsynthetase	16.95769119	0.167066667	4	4	21254034.76	1012096.894
MGG_07258	U3smallnucleolarribonucleoproteinmpp10	28.7956422	0.1095	7	8	33750844.89	1250031.292
MGG_12336	patternformationproteinEMB30	26.01867715	0.0527	7	8	38355819.45	456616.8982
MGG_11039	N-acetyltransferase10	122.5694173	0.379933333	27	35	120858915.5	2158194.92
MGG_00146	hypotheticalprotein	29.8093611	0.253366667	7	10	241362397.6	10056766.57
MGG_04100	STE/STE11/CDC15proteinkinase	4.725217904	0.0074	1	2	159638.9837	1900.464091
MGG_00336	COP9signalosomecomplexsubunit4	2.783936758	0.0294	1	1	8328777.944	308473.2572
MGG_08008	Formyltetrahydrofolatedeformylase	4.902300803	0.0716	1	2	9528042.752	595502.672

MGG_06848	hypotheticalprotein	5.072348799	0.0294	1	1	1303863.603	37253.24581
MGG_02489	branched-chain-amino-acidaminotransferase	12.62867348	0.123766667	3	5	34010342.31	1619540.11
MGG_11188	hypotheticalprotein	19.79669762	0.072566667	5	6	9001888.348	272784.4954
MGG_16793	actin-likeprotein	13.90686261	0.124233333	4	4	12639988.2	574544.9181
MGG_04843	Fungal-specificZn(2)-Cys(6)domain-containingtranscrip tionfactor,putative	14.72043081	0.064466667	3	4	10051742.48	335058.0826
MGG_04040	DEAHboxpolypeptide37	21.17661326	0.051466667	5	5	47972280.59	773746.4611
MGG_09562	hypotheticalprotein	10.4980046	0.067566667	2	2	4272321.172	101721.9327
MGG_09282	hypotheticalprotein	14.4962337	0.0525	3	3	4888460.925	81474.34875
MGG_04389	replicationfactorCsubunit2	7.80925657	0.083333333	2	3	3585542.663	123639.4022
MGG_02460	ribosomebiogenesisproteinYTM1	59.69940968	0.4029	15	17	193370347	6445678.234
MGG_17000	adenylosuccinatesynthetase	5.145426165	0.035466667	1	1	2501413.643	83380.45475
MGG_04477	DNA-directedRNApolymeraseIIIsubunitRPC1	4.215223875	0.007533333	1	2	873608.6017	9293.708528
MGG_00167	hypotheticalprotein	28.88325218	0.2798	7	7	85869566.39	4519450.863
MGG_11573	30SribosomalproteinS10	23.92613983	0.259266667	6	8	43160434.72	2158021.736

MGG_00446	caseinkinaseIIsubunitbeta-1	38.35283211	0.345666667	9	15	114849397.5	6755846.909
MGG_01826	pre-mRNA-splicingfactorcwc26	29.09468053	0.3343	7	9	12409015.39	620450.7696
MGG_01183	pescadillo	27.62071622	0.098566667	7	9	12509280.49	481126.1727
MGG_09464	hypotheticalprotein	16.70529737	0.175366667	5	6	4763714.33	226843.5395
MGG_00748	myosin-1	15.54445104	0.053366667	4	4	54752274.81	701952.2411
MGG_07126	hypotheticalprotein	27.77229275	0.139666667	7	10	11523462.11	460938.4845
MGG_03579	hypotheticalprotein	23.15403867	0.118766667	6	7	15733661.22	524455.3741
MGG_03252	E3ubiquitin-proteinligaseptr1+RNAtransporter1	65.87753186	0.053933333	17	18	44495610.4	205998.1963
MGG_10236	hypotheticalprotein	4.074610105	0.015266667	1	1	11151591.9	146731.4724
MGG_11724	hypotheticalprotein	5.948859822	0.065333333	2	2	2625078.318	154416.3717
MGG_08746	ste/ste20/yskproteinkinase	2.841813587	0.0099	1	1	157077.3594	3831.155106
MGG_16743	hypotheticalprotein	14.66820104	0.086333333	4	4	8902575.365	228271.1632
MGG_06263	hypotheticalprotein	5.072348799	0.0698	1	1	581158.3967	52832.58152
MGG_08318	oxidoreductaseucpA	5.585024064	0.049233333	2	2	5848615.136	417758.224

MGG_00960	phospholipaseD1	4.129815547	0.0067	1	1	300937.2185	3271.056723
MGG_05994	hypotheticalprotein	61.97722412	0.145733333	16	18	32146176.84	387303.3354
MGG_03733	ribonucleaseP/MRPproteinsubunitRPP1	14.65941854	0.200633333	4	5	24470917.61	1439465.742
MGG_06346	ribosomebiogenesisproteinMAK21	114.020742	0.359166667	25	36	107977454.8	2117204.996
MGG_08140	AdoMet-dependentrRNAmethyltransferasespb1	13.72055408	0.058966667	3	4	14551252.53	354908.5984
MGG_09181	hydroxymethylglutaryl-CoAlyase	31.23607045	0.386233333	7	11	38706081.96	2419130.123
MGG_06681	arginine-tRNA-proteintransferase1	10.58257231	0.065933333	3	3	5049831.306	219557.8829
MGG_08323	mitochondriallargeribosomalsubunit	17.84841121	0.151133333	4	4	24889782.51	1185227.738
MGG_01016	chaperonednaJ6	3.324148009	0.042166667	1	1	3216149.391	178674.9662
MGG_03517	hypotheticalprotein	21.77494928	0.089166667	5	5	48901976.42	1481878.073
MGG_04023	hypotheticalprotein	18.96013006	0.2312	5	7	17900957.12	1193397.141
MGG_07737	U3smallnucleolarRNA-associatedprotein20	71.85401104	0.0795	18	18	87890554.4	614619.2615
MGG_15853	hypotheticalprotein	7.247389753	0.066833333	2	2	2124841.317	92384.40509
MGG_04179	ATP-dependentRNAhelicaseDBP10	16.62229821	0.056166667	4	4	38551831.21	755918.2589

MGG_05260	hypotheticalprotein	33.90362325	0.072633333	9	10	19822049.26	198220.4926
MGG_10686	hypotheticalprotein	25.21865799	0.096233333	6	7	13955983.81	465199.4602
MGG_10334	mannitol2-dehydrogenase	9.192276735	0.0498	2	2	2717333.868	84916.68338
MGG_17698	hypotheticalprotein	2.618603556	0.0049	1	1	1230003.303	14819.3169
MGG_01893	ribonucleasePproteinsubunitp29	11.09211047	0.1276	3	4	77712278.27	4317348.793
MGG_02514	ankyrinrepeatproteinnuc-2	5.072348799	0.0125	1	1	755646.968	15742.64517
MGG_06686	hypotheticalprotein	9.417823143	0.038533333	2	2	4724370.99	104986.022
MGG_04613	ATP-bindingdomain-containingprotein3	9.002956751	0.089	3	3	5225195.098	237508.8681
MGG_06600	carbonatedehydratase	11.48939631	0.2154	3	3	6222576.462	622257.6462
MGG_05694	Gtr1/RagAGdomain-containingprotein,Gtr1/RagAGdomain-containingprotein,variant	13.85625376	0.0922	3	3	32769615.03	1724716.58
MGG_08841	hypotheticalprotein	11.9655827	0.033133333	3	4	3075640.215	51260.67025
MGG_08173	NADP-dependentmalicenzyme	7.766112423	0.041866667	2	4	6690288.925	176060.2349
MGG_15582	rRNA-processingproteinEFG1	5.072348799	0.048	1	2	1411402.183	100814.4416
MGG_06071	50SribosomalproteinL11	7.380056083	0.161333333	2	2	933244.5884	71788.04526

MGG_08691	30SribosomalproteinS12	7.69039955	0.201166667	3	3	2703010.436	245728.2214
MGG_04470	hypotheticalprotein	26.62112032	0.1059	7	8	22781686.96	414212.4901
MGG_04856	proteintransporterSEC61subunitalpha	6.600326844	0.0469	2	2	3508966.286	194942.5714
MGG_14751	FACTcomplexsubunitpob-3	93.47259624	0.4694	21	30	116175131.9	4006039.031
MGG_01410	glutathioneS-transferaseGst3	4.932320758	0.042	2	2	1225656.126	45394.67133
MGG_07121	DNAmismatchrepairproteinMutL	2.624283244	0.013	1	1	252815.0247	5495.978798
MGG_03115	hypotheticalprotein	4.725217904	0.0258	1	1	954538.0074	34090.64312
MGG_11141	lonproteaselikeprotein	15.46405959	0.0453	4	6	10495325.63	174922.0938
MGG_02598	30SribosomalproteinS16	16.37172827	0.454033333	4	7	18346585.08	2620940.725
MGG_06180	hypotheticalprotein	4.725217904	0.031133333	1	1	3832956.202	136891.2929
MGG_11144	chaperoneDnaJ	19.24103434	0.1203	5	5	6195348.073	193604.6273
MGG_06691	4-hydroxyphenylpyruvatedioxygenase	4.960942736	0.029433333	1	1	48627146.28	2210324.831
MGG_17371	hypotheticalprotein	25.3137757	0.196866667	6	8	448743405.7	17949736.23
MGG_09313	ribosomebiogenesisproteinERB1	62.28905088	0.293	14	21	49430016.95	1149535.278

MGG_03893	pre-mRNA-splicingfactorATP-dependentRNAhelicasePRP43	32.39544297	0.1335	9	10	24229356.87	550667.2016
MGG_11801	AP-1complexsubunitgamma-1	14.20493678	0.0509	4	4	5185105.131	123454.8841
MGG_02467	ribonucleoprotein-associatedprotein	8.24421483	0.2381	2	3	6042870.556	1208574.111
MGG_08117	DRAPdeaminase	5.287765578	0.030133333	1	1	4086182.278	163447.2911
MGG_06995	37SribosomalproteinS5	64.58063836	0.4369	16	20	80143368.18	2585269.941
MGG_02992	hypotheticalprotein	7.729487027	0.0458	2	2	4726720.35	152474.85
MGG_04465	hypotheticalprotein	3.897371168	0.0232	1	1	2241875.124	160133.9374
MGG_00447	brefeldinAresistanceprotein	7.270964104	0.0139	2	2	2369351.972	32018.2699
MGG_03154	hypotheticalprotein	2.978494071	0.0204	1	1	2402083.95	60052.09875
MGG_09645	Polyketidesynthase	26.71172522	0.052666667	7	7	15926141.94	151677.5423
MGG_11383	Phosphoacetylglucosaminemutase	6.832180746	0.047066667	2	2	4588400.526	134952.9566
MGG_04802	periodictryptophanprotein1	46.84035475	0.2644	10	13	81304747.48	3252189.899
MGG_08049	ATP-dependentRNAhelicaseDBP4	19.53887493	0.065966667	5	5	9358810.278	207973.5617
MGG_01520	hypotheticalprotein	62.95656068	0.395666667	14	18	49094879.96	1963795.198

MGG_12862	phosphatidylinositol4-kinasePIK1alpha	4.508211923	0.019566667	2	2	4542616.371	89070.90924
MGG_04506	26Sproteasomenon-ATPaseregulatorysubunit4	7.950453755	0.105433333	2	3	4601524.413	306768.2942
MGG_01324	urease	16.1812527	0.079066667	4	4	122154599	3301475.65
MGG_14872	calpain-9	13.43681709	0.0594	4	5	5029984.226	125749.6057
MGG_00977	U3smallnucleolarribonucleoproteinIMP4	24.75754155	0.318633333	6	8	16228721.12	811436.0561
MGG_07006	hypotheticalprotein	22.32910772	0.2324	5	7	65874472.85	4117154.553
MGG_10565	zuotin	3.868741144	0.0308	2	2	4128193.03	158776.655
MGG_04652	DNA-directedRNApolymeraseIIlargestsubunit	56.47920872	0.122766667	15	18	104409999.5	1160111.105
MGG_03684	mitochondrialdistributionandmorphologyprotein38	4.596079965	0.0266	1	1	591427.3885	21122.40673
MGG_07600	hypotheticalprotein	4.443994815	0.0335	1	1	381211.6826	10891.76236
MGG_17504	ironsulfurassemblyprotein1	5.072348799	0.0496	1	1	71982.87794	3788.572523
MGG_01012	ISWIchromatin-remodelingcomplexATPaseISW2	6.830746423	0.0189	2	2	823143.1832	11759.18833
MGG_05250	hypotheticalprotein	3.586751821	0.0163	1	1	906332.5627	21077.50146
MGG_03841	E3ubiquitin-proteinligaseUPL3	5.174488352	0.017133333	2	2	2764128.069	30375.03373

MGG_05268	uspdomain-containingprotein	16.81847324	0.1654	4	4	6119333.102	322070.1633
MGG_08640	GTP-bindingprotein1	33.0993698	0.283033333	8	9	39167531.46	1780342.339
MGG_05384	ankyrinrepeatdomain-containingprotein29	3.700013412	0.0128	1	1	43297.40935	562.3040175
MGG_06162	DNAtopoisomerase2	43.73725	0.082833333	11	14	76639566.34	798328.8161
MGG_07201	mitochondrialDNAreplicationproteinYHM2	5.79454089	0.076733333	1	1	684731.3453	38040.6303
MGG_01595	eukaryotictranslationinitiationfactor3subunitM	147.6868671	0.6319	30	106	1052273193	47830599.67
MGG_01000	smallnucleolarribonucleoproteincomplexsubunitUtp14	17.9296253	0.058	5	5	9937429.178	248435.7294
MGG_00665	tyrosyl-tRNAsynthetase	22.11571442	0.1031	6	6	13269773.82	340250.6107
MGG_07593	CytochromeP450monooxygenase	5.488689279	0.0361	1	1	4063265.364	176663.7115
MGG_00529	peroxisomalbiogenesisfactor6	19.26884501	0.060133333	6	6	64004639.56	927603.4718
MGG_03329	smallheatshockprotein	5.665909379	0.082533333	1	1	2348301.657	156553.4438
MGG_07175	hypotheticalprotein	15.45300167	0.1373	4	5	17056353.54	568545.1179
MGG_04449	branched-chainalpha-ketoacidlipoamideacyltransferase	8.172225794	0.049033333	2	2	800795.964	29659.10978
MGG_06202	GTPbindingproteinBms1	58.56156065	0.1495	15	16	52206773.01	828678.9367

MGG_01404	NAD(P)H-dependentD-xylosereductase	4.713416866	0.061	1	1	2016426.55	87670.71958
MGG_06554	hypotheticalprotein	6.524018285	0.028166667	2	2	3695196.862	142122.9562
MGG_04994	plasmamembraneH+-ATPase	7.506374354	0.025233333	2	2	2517814.255	52454.46364
MGG_05856	ankyrinrepeatandSOCSboxprotein7	8.728321877	0.0197	2	2	5881926.503	70866.58438
MGG_02617	2-methylcitratesynthase	14.25122418	0.128566667	3	3	4661232.738	179278.1822
MGG_04377	rho-typeGTPase-activatingprotein1	27.62025824	0.0655	6	6	7074935.981	101070.514
MGG_09300	DNAreplicationlicensingfactormcm7	41.50293612	0.170566667	10	13	215144332.3	4059327.024
MGG_17982	phosphopantothenate-cysteineligase	17.54864206	0.135166667	4	5	14474181.07	723709.0536
MGG_16520	hypotheticalprotein	4.928346527	0.0386	2	2	972158.0622	33522.6918
MGG_05156	eukaryotictranslationinitiationfactor3subunitH	109.4068157	0.7355	23	83	696944522.1	38719140.12
MGG_00881	DNAprimasesmallsubunit	5.408612951	0.030233333	2	2	5418181.905	150505.0529
MGG_06643	hypotheticalprotein	7.223819616	0.031633333	2	2	2830731.433	57770.02925
MGG_05170	54SribosomalproteinL17	39.92968237	0.319866667	10	12	36318808.57	1345141.058
MGG_04150	AP-2complexsubunitbeta	10.09592234	0.036366667	3	3	688184.5594	16385.34665

MGG_03229	ATP-dependentRNAhelicasedbp6	38.8801444	0.141733333	9	10	25190833.25	514098.6378
MGG_01469	homoserineO-acetyltransferase	3.43675139	0.022933333	1	1	566903.7963	21803.99217
MGG_12154	Fattyacidsynthasesubunitalpha	42.45365415	0.071166667	11	11	66972297.29	608839.0662
MGG_03160	aldehydereductase1	2.120573465	0.0193	1	1	1054876.234	47948.91972
MGG_01722	adenylylcyclase-associatedprotein	11.39988781	0.065666667	3	3	3930786.609	135544.3658
MGG_12764	hypotheticalprotein	4.303336147	0.098766667	1	1	3280350.482	656070.0963
MGG_01562	hypotheticalprotein	23.55494513	0.1816	6	7	22867414.18	914696.5674
MGG_00449	eukaryoticpeptidechainreleasefactorGTP-bindingsubunit	6.840179822	0.035566667	2	2	8307348.441	197794.0105
MGG_06524	Fructose-2,6-bisphosphatase	23.66567488	0.158	6	6	12219587.7	359399.6383
MGG_15868	Argininosuccinatesynthase	15.15293536	0.1036	4	5	2590041.225	112610.488
MGG_10651	50SribosomalproteinL17	10.99921321	0.155033333	3	3	4292546.288	286169.7525
MGG_07074	Cystathioninebeta-lyase	5.587430319	0.0411	1	2	4325646.387	180235.2661
MGG_00979	mitochondrialimportreceptorsubunittom-70	12.34894548	0.0595	3	3	6997518.73	189122.1278
MGG_03238	zincfingerproteinZPR1	19.87667577	0.105266667	5	5	9493193.756	379727.7502

MGG_07301	hypotheticalprotein	3.806415543	0.0467	1	1	1374777.895	85923.61843
MGG_02838	hypotheticalprotein	32.22666368	0.111433333	9	11	55560165.27	910822.3815
MGG_07298	deoxycytidylatedeaminase	12.13665733	0.127766667	3	3	10484348.39	524217.4196
MGG_12749	Glutathionereductase	8.018732725	0.057766667	2	2	14190749.55	473024.9849
MGG_08708	WDrepeat-containingprotein	15.71938885	0.102033333	3	4	47292468.89	1351213.397
MGG_02384	mannosyltransferase	2.642161518	0.0142	1	1	346873.3121	13874.93249
MGG_08643	PEK/GCN2proteinkinase	17.41221624	0.0357	4	5	6964940.839	71070.82489
MGG_05806	hypotheticalprotein	13.20638355	0.0591	3	3	5449375.818	175786.3167
MGG_04825	endopolyphosphatase	8.682741166	0.051366667	2	2	1176688.034	35657.21316
MGG_16039	hypotheticalprotein	5.143999165	0.100533333	1	2	639010.7301	91287.24716
MGG_09556	mitochondrial37SribosomalproteinS17	17.2842928	0.439833333	4	5	14888661.89	1353514.717
MGG_08015	hypotheticalprotein	4.635920736	0.0557	1	1	134317.41	11193.1175
MGG_06270	Adenosinekinase	7.308122334	0.0997	2	2	2432148.464	128007.8139
MGG_01004	hypotheticalprotein	7.541340125	0.0606	2	2	2296918.816	143557.426

MGG_01551	long-chain-fatty-acid-CoAligase1	8.843186959	0.055133333	2	3	213161.3373	6876.17217
MGG_06110	ATP-bindingdomain1familymemberB	5.599851058	0.048533333	1	1	7709565.978	453503.8811
MGG_06429	hypotheticalprotein	12.48051433	0.084033333	3	3	2789208.259	84521.46239
MGG_17683	hypotheticalprotein	19.67875018	0.062233333	5	7	8022936.162	151376.154
MGG_04200	hypotheticalprotein	4.831855801	0.1106	1	2	443499.79	36958.31584
MGG_06381	hypotheticalprotein	10.66261863	0.060266667	2	2	11202289.9	386285.8586
MGG_01561	nucleolarGTP-bindingprotein2	36.59079182	0.175066667	9	10	149016910.6	3725422.765
MGG_08829	transcriptionalrepressorrco-1	27.77559763	0.1587	7	10	27748143.53	816121.8687
MGG_06969	minichromosomelossprotein1	7.204651861	0.0293	2	2	1662013.322	35361.98558
MGG_03186	1,4-alpha-glucan-branchingenzyme	8.381521688	0.036633333	2	3	2142140.609	54926.68228
MGG_05849	hypotheticalprotein	4.953411781	0.083033333	2	2	1984577.129	124036.0706
MGG_12633	hypotheticalprotein	5.072348799	0.0437	1	1	1295147.143	68165.63912
MGG_00149	serine/threonine-proteinphosphatasePP-Z	5.374860053	0.041533333	1	2	1071330.17	41205.00655
MGG_03027	BTB/POZdomain-containingprotein3	4.575492164	0.0285	1	1	1101708.897	28248.94608

MGG_06465	hypotheticalprotein	18.1740584	0.050633333	5	5	8600429.245	103619.6295
MGG_03203	54SribosomalproteinL16	5.072348799	0.0438	1	1	498676.8638	33245.12426
MGG_14800	hypotheticalprotein	44.91171746	0.244133333	12	13	27359956.47	829089.59
MGG_04331	37SribosomalproteinMRP4	39.04640334	0.3124	9	14	45308190	1510273
MGG_03928	37SribosomalproteinS25	30.45700301	0.3063	8	11	46096036.88	2560890.938
MGG_04083	smallnuclearribonucleoproteinSmD3	7.249293	0.154066667	2	2	6837686.921	854710.8651
MGG_04985	hypotheticalprotein	5.231898149	0.032666667	2	2	2212161.265	50276.39239
MGG_03945	Acetolactatesynthase	22.90851951	0.1204	5	6	10406832.01	385438.2225
MGG_07433	hypotheticalprotein	18.24534401	0.1452	5	6	15779395.24	751399.7735
MGG_05970	hypotheticalprotein	21.69078047	0.227733333	5	7	10258009	539895.2105
MGG_12159	Uridinekinase	10.2277939	0.060233333	3	3	4940190.013	176435.3576
MGG_08661	acyl-CoAdehydrogenasefamilymember11	3.413356484	0.024333333	1	1	1150502.614	35953.20668
MGG_00053	hypotheticalprotein	21.95490585	0.082833333	5	6	7116639.273	197684.4243
MGG_11377	mitochondrial37SribosomalproteinS8	33.22701257	0.639733333	8	12	36454705.52	3037892.127

MGG_06379	midasin	6.634327407	0.006033333	2	2	65150.30398	247.719787
MGG_03720	hypotheticalprotein	7.485426724	0.0693	2	2	1204349.228	63386.80148
MGG_13200	eukaryotictranslationinitiationfactor2subunitalpha	34.02826357	0.4043	9	10	70278553.53	3513927.677
MGG_09830	AAAfamilyATPase	12.98696782	0.034733333	3	3	6277152.309	108226.7639
MGG_03572	hypotheticalprotein	21.49979424	0.5512	5	6	15683829.88	1960478.735
MGG_04617	hypotheticalprotein	6.75825118	0.0103	1	1	1288315.185	18671.23456
MGG_03215	DNA-directedRNApolymeraseIIsubunitRPB3	16.2316395	0.176766667	4	4	11836758.3	696279.9
MGG_03696	CMGC/CK2proteinkinase	66.09962372	0.497566667	15	28	193302647.5	8786483.979
MGG_08561	RINGfingerandCHYzincfingerdomain-containingprotein	8.08355065	0.0395	2	2	1308503.315	26704.14929
MGG_16744	hypotheticalprotein	84.83876288	0.279266667	20	28	70609648.49	1332257.519
MGG_04862	septum-promotingGTP-bindingprotein1	6.951295426	0.0654	2	2	1383311.238	98807.94557
MGG_14801	IdgAdomain-containingprotein	4.10641964	0.0116	1	1	264469.4804	6450.475132
MGG_03220	isoleucyl-tRNAsynthetase	6.551210424	0.0232	2	2	2176479.88	38183.85754
MGG_04112	hypotheticalprotein	40.4980213	0.357966667	8	12	34203091.61	1425128.817

MGG_08694	hypotheticalprotein	3.403309111	0.064866667	1	1	0	0
MGG_00795	hypotheticalprotein	3.192587809	0.0056	1	1	1038142.061	15729.42516
MGG_03661	Phosphoserinephosphatase	9.285616484	0.099866667	2	2	8505818.661	500342.2742
MGG_04857	tRNAisopentenyltransferase	5.866566291	0.053266667	2	2	4871706.46	147627.4685
MGG_09508	hypotheticalprotein	29.12820262	0.297766667	6	9	38181442.99	2545429.532
MGG_15229	Rpp14familyprotein	6.795263267	0.097766667	1	1	2903879.102	290387.9102
MGG_01114	NADP-dependentmannitoldehydrogenase	10.94556721	0.082633333	3	3	4398847.594	209468.9331
MGG_01045	arrestindomain-containingprotein	16.79247844	0.0885	5	5	20168975.58	650612.1155
MGG_04724	anaphase-promotingcomplexsubunit2	3.197657553	0.008633333	1	1	4669455.501	79143.31357
MGG_09306	DNAmismatchrepairproteinMSH3	3.232262338	0.008433333	1	1	2967451.205	44290.31649
MGG_06734	hypotheticalprotein	4.829236647	0.062	1	1	2321023.397	145063.9623
MGG_06049	ribosomalRNAassemblyproteinKRR1	13.83730437	0.1448	3	4	10807676.01	675479.7506
MGG_01078	ribosomebiogenesisproteinBRX1	56.80147838	0.5495	13	26	123081027.5	6154051.373
MGG_00560	ATP-dependentRNAhelicaseMAK5	16.79737125	0.088633333	5	5	11508013.67	250174.2103

MGG_03291	SDA1domain-containingprotein	32.71609247	0.1419	8	10	13594347	323674.9285
MGG_06322	brixdomain-containingprotein	21.79810398	0.160033333	6	6	23901733.07	995905.5444
MGG_10526	hypotheticalprotein	50.49309262	0.089766667	12	14	30303880.27	333009.6733
MGG_06960	hypotheticalprotein	3.534618212	0.0118	1	1	3048557.427	84682.15076
MGG_00685	replicationfactor-Aprotein1	7.06878813	0.037733333	2	2	2160713.402	51445.55718
MGG_06321	glycyl-tRNAsynthetase	6.84418211	0.0349	2	2	2198174.583	56363.45084
MGG_06904	hypotheticalprotein	72.07698051	0.3805	17	28	93389570.3	2334739.258
MGG_04405	CCCHzincfingerandSMRdomain-containingprotein	5.211008183	0.025766667	1	1	1272975.558	32640.39893
MGG_01100	smallnucleolarribonucleoproteincomplexsubunit	41.88384272	0.2506	10	12	50283300.39	1622041.948
MGG_09573	mitochondrial37SribosomalproteinS19	8.62722629	0.229866667	2	2	3835808.239	479476.0299
MGG_04996	nucleolarcomplex-associatedprotein3	54.4668563	0.250633333	12	16	47776893.21	1365054.092
MGG_06856	hypotheticalprotein	12.7746881	0.107433333	3	3	4005225.846	190725.0403
MGG_15156	phosphatidylinositol3-kinasetor2	14.98894623	0.016933333	4	4	5823769.384	38568.00916
MGG_05204	transcriptioninitiationfactorTFIIDsubunit14	5.072348799	0.0746	1	1	644703.7287	49592.59451

MGG_01165	isoleucyl-tRNAsynthetase	14.25253808	0.050633333	4	5	16808028.18	317132.6072
MGG_06511	2-oxoisovaleratedehydrogenasesubunitbeta	2.128311	0.0199	1	1	23062.48723	1213.815117
MGG_04956	hypotheticalprotein	6.166310461	0.029033333	2	2	5121715.744	134781.9933
MGG_11063	nucleolarprotein12	7.861425833	0.056833333	2	2	11654063.58	485585.9826
MGG_06500	WDrepeat-containingprotein	34.254933	0.191833333	8	11	19118931.8	579361.5696
MGG_10252	hypotheticalprotein	14.53292827	0.106333333	3	4	7611375.375	245528.2379
MGG_00220	NADP-dependentalcoholdehydrogenase6	4.9695418	0.064833333	2	2	7668830.88	479301.93
MGG_07916	vacuolarproteinsorting-associatedprotein4	3.355089557	0.026533333	1	1	1963987.817	65466.26058
MGG_00363	hypotheticalprotein	22.76638857	0.050666667	5	5	8572017.21	114293.5628
MGG_14574	hypotheticalprotein	34.14017941	0.198733333	8	9	11274714.55	304722.015
MGG_09285	hypotheticalprotein	21.03919639	0.164633333	6	6	17616825.12	629172.3259
MGG_11205	dyneinheavychain	19.57180931	0.0125	5	5	3538377.755	13105.1028
MGG_09647	Acyl-CoAsynthetase	8.194828835	0.0462	2	2	1888964.516	78706.85483
MGG_17514	hypotheticalprotein	43.91371071	0.2407	11	12	65285109.64	2510965.755

MGG_02603	hypotheticalprotein	4.560953304	0.060633333	2	2	1045806.07	74700.43358
MGG_02476	hypotheticalprotein	33.35679397	0.044933333	8	10	9945086.499	83572.15545
MGG_00500	meioticallyup-regulated71protein	10.06502193	0.041	2	2	3881726.074	102150.6861
MGG_09301	hypotheticalprotein	48.148492	0.279633333	11	15	100458620	3720689.629
MGG_06261	eukaryotictranslationinitiationfactor3135kDasubunit	266.8073424	0.5126	56	98	585196943.4	8734282.737
MGG_03859	vacuolarimportanddegradationprotein27	11.84612943	0.057666667	3	4	4484663.659	104294.5037
MGG_05484	AP-2complexsubunitmu-1	8.122667294	0.0671	2	2	4439919.812	138747.4941
MGG_08914	hypotheticalprotein	4.697757239	0.013333333	1	1	2029623.349	29414.83114
MGG_00650	hypotheticalprotein	4.214486259	0.0585	1	1	87179.65171	8717.965171
MGG_11568	DNAhelicase	9.484597765	0.069666667	2	3	2149904.37	52436.69194
MGG_06320	ste/ste20/pakaproteinkinase	26.66128926	0.099733333	7	7	26276742.39	547432.1332
MGG_13500	U5smallnuclearribonucleoproteincomponent	79.99938426	0.291533333	20	23	48865566.76	828229.9451
MGG_14763	Cullin-4B	21.52812893	0.078733333	7	7	8590269.421	153397.6682
MGG_07728	hypotheticalprotein	71.82490949	0.2219	18	22	39524981.96	745754.3765

MGG_05146	ribosomalRNA-processingprotein1	17.53028459	0.180766667	4	4	19746795.24	897581.602
MGG_04478	fimbrin	4.407667518	0.021033333	1	1	1788073.335	38871.15945
MGG_07098	condensinsubunit	19.3847647	0.048366667	5	5	304034485.8	4406296.896
MGG_10180	pre-mRNA-splicingfactorcwc22	35.71979474	0.115766667	9	11	118805111.6	2527768.331
MGG_01123	pre-rRNAprocessingproteinRrp12	4.234496819	0.0108	1	1	981975.1214	15838.30841
MGG_10327	hypotheticalprotein	210.5038881	0.6246	48	146	1581746345	36784798.72
MGG_01759	DNAreplicationlicensingfactormcm3	13.6333906	0.054666667	4	4	4182264.309	77449.33905
MGG_02511	50SribosomalproteinL3	22.15431686	0.2062	6	7	42341247.62	1693649.905
MGG_16395	ATP-dependentZnprotease	15.650706	0.109033333	4	5	3808802.736	146492.4129
MGG_11604	hypotheticalprotein	6.018468702	0.0365	2	2	2254268.935	86702.65133
MGG_08122	DNAreplicationlicensingfactorMCM2	58.55511945	0.217633333	14	17	53340724.15	969831.3483
MGG_02944	HALproteinkinase	5.666051789	0.023533333	1	1	3074827.819	76870.69547
MGG_03317	eukaryotictranslationinitiationfactor3subunitE	131.6743205	0.6839	27	113	1225174778	51048949.07
MGG_07109	Eukaryotictranslationinitiationfactor3subunitG	69.04475007	0.5329	17	40	412570846.7	24268873.33

MGG_04674	Fungal-specificZn(2)-Cys(6)domain-containingtranscriptionfactor,putative	15.55221783	0.054666667	4	5	9576528.711	290197.8397
MGG_01007	hypotheticalprotein	12.55457867	0.165966667	3	4	5162444.719	303673.2187
MGG_05033	Fungal-specificZn(2)-Cys(6)domain-containingtranscriptionfactor,putative	3.847177953	0.0202	1	1	1115386.104	32805.47364
MGG_10653	eukaryotictranslationinitiationfactor3subunitF	102.331391	0.7	21	83	1110046412	61669245.11
MGG_04013	smallnuclearribonucleoproteinSmD2	8.134034753	0.242166667	2	3	16888959.87	2412708.552
MGG_00170	proteasomecomponentPRE2	4.118084664	0.0352	1	1	3021522.39	167862.355
MGG_06935	40SribosomalproteinS9	30.93866805	0.238866667	8	13	24360289.26	1059143.011
MGG_00758	geranylgeranylpyrophosphatesynthase	8.458795217	0.062333333	2	2	2748198.804	119486.9045
MGG_04118	fattyacidsynthasebetasubunitdehydratase	111.361913	0.207866667	28	34	64772392.05	535309.0252
MGG_02570	dienelactonehydrolase	4.402739606	0.0375	2	2	93276.53459	4239.842481
MGG_07291	CMGC/CDK/CRK7proteinkinase	8.481165648	0.026166667	2	3	919187.2421	14825.60068
MGG_09302	Ribosomebiogenesisprotein	18.02513351	0.336766667	4	7	9142042.624	1306006.089
MGG_04536	hypotheticalprotein,hypotheticalprotein,variant	4.704239253	0.017733333	2	2	3185282.913	65005.77374

MGG_07035	epsilon-COP	8.614884813	0.106633333	2	3	41229853.37	2576865.836
MGG_00466	celldivisioncontrolprotein42	6.545327792	0.1357	2	3	6551009.326	727889.9251
MGG_08302	rRNA-processingproteinFCF1	11.22625895	0.233833333	3	3	11265106.42	1251678.491
MGG_00859	ariadne-1	15.028258	0.075333333	4	5	14262230.02	491801.0352
MGG_04741	smallnuclearribonucleoproteinSmD1	6.09115749	0.221333333	2	2	5675049.857	1418762.464
MGG_04425	phenylalanyl-tRNAsynthetasesubunitalpha	8.413937636	0.0618	2	3	2876182.658	95872.75526
MGG_10575	hypotheticalprotein	6.049856834	0.053833333	1	2	303066.2745	21647.59103
MGG_04978	E3ubiquitinligasecomplexSCFsubunitscon-3	7.890884496	0.1488	2	2	833635.4719	75785.0429
MGG_00840	hypotheticalprotein	20.9348237	0.1175	6	7	34092566.04	1704628.302
MGG_10160	ribosomebiogenesisproteinRLP24	9.266668069	0.0684	2	3	9375230.154	1171903.769
MGG_02493	hypotheticalprotein	6.310702856	0.020533333	1	1	100871530.5	1939837.125
MGG_11360	nucleolarcomplexprotein4	20.24910146	0.137766667	5	6	10106512.28	360946.8671
MGG_04091	hypotheticalprotein	28.79559068	0.224833333	7	7	23130692.22	1285038.457
MGG_13203	ATPase	20.69944764	0.084866667	5	5	69033955.77	1643665.614

MGG_06395	intron-bindingproteinaquarius	23.08142596	0.050333333	7	7	15507706.52	198816.7502
MGG_07316	FACTcomplexsubunitspt-16	187.4649186	0.460633333	43	62	210779147.2	3512985.786
MGG_05244	pre-mRNA-processing-splicingfactor8	61.38825641	0.083166667	16	17	376065893.6	2611568.706
MGG_06480	40SribosomalproteinS12	47.40651563	0.6122	12	27	106918574.3	11879841.59
MGG_06984	DNA-directedRNApolymeraseIIIsubunitRPC2	6.159826127	0.0256	2	2	804703.7883	11333.85617
MGG_06470	DNArepairhelicaserad25	28.87857399	0.115766667	7	8	17051841.89	341036.8378
MGG_03302	hypotheticalprotein	88.16515605	0.430766667	20	35	326062135	9590062.793
MGG_11196	originrecognitioncomplexsubunit4	5.491466647	0.0243	1	1	6465440.486	150359.0811
MGG_07465	periodictryptophanprotein2	61.1018503	0.218133333	15	23	74814374.13	1626399.438
MGG_09393	glutaminyl-tRNAsynthetase	5.93365731	0.018433333	1	1	73117.59023	2031.044173
MGG_11916	CAP20	13.30573138	0.240133333	3	3	6378658.429	637865.8429
MGG_07833	ferricreductase	24.50698673	0.107933333	6	7	17026136.69	532066.7714
MGG_07928	ubiquitin-40SribosomalproteinS27a	37.76807001	0.532466667	10	17	29054564.15	2641324.014
MGG_04995	U3smallnucleolarRNA-associatedprotein10	82.1364283	0.133266667	20	22	89553022.85	962935.7296

MGG_06189	hypotheticalprotein	9.003905568	0.071766667	3	3	2511083.181	147710.7754
MGG_09896	hypotheticalprotein	3.689289816	0.028066667	1	1	1993899.3	71210.68928
MGG_04641	transcriptionfactorAATF/Che-1	45.15211748	0.2203	11	13	28174960.87	1408748.044
MGG_10199	leucineRichRepeatdomain-containingprotein	11.25724057	0.048866667	3	3	2209346.881	43320.52709
MGG_05295	hypotheticalprotein	13.38807894	0.143766667	3	3	4591016.209	208682.5549
MGG_00976	regulator-nonsensetranscripts1	18.59353141	0.058533333	5	5	6288901.745	96752.33453
MGG_01106	dDENNdomain-containingprotein	16.57713472	0.0557	4	4	9096207.885	144384.2521
MGG_02734	hypotheticalprotein	3.900214076	0.0221	1	1	7456318.886	257114.4443
MGG_03537	importinsubunitbeta-3	8.81421221	0.030366667	2	2	24698968.56	493979.3711
MGG_08159	ATPNADkinase	6.75825118	0.047066667	1	2	209753.7139	6169.226881
MGG_01671	eukaryotictranslationinitiationfactor6	20.32640728	0.332	4	5	15275017.13	1527501.713
MGG_10380	cystathionine-gamma-lyase	6.071199468	0.055533333	1	2	1688109.089	99300.53467
MGG_06786	disulfide-isomeraseA6precurso	7.250329918	0.0474	2	2	2485446.675	80175.69921
MGG_13188	voltage-gatedpotassiumchannelsubunitbeta-2	7.688129051	0.0826	2	2	17200850.32	747863.0575

MGG_04106	hypotheticalprotein	34.53366382	0.290966667	8	10	22401046.42	746701.5474
MGG_12175	gramicidinSsynthetase1	14.48873018	0.0106	4	4	3776439.696	15351.38088
MGG_10126	Aspartylaminopeptidase	5.100570581	0.026166667	1	1	1148179.128	33769.97437
MGG_08178	U3smallnucleolarRNA-associatedprotein13	82.00904905	0.301866667	19	21	35656479.91	713129.5981
MGG_00143	hypotheticalprotein	25.26149039	0.216866667	6	7	13919637.08	605201.6123
MGG_00613	hypotheticalprotein	19.71883417	0.1432	4	14	94431536.97	5554796.292
MGG_02458	hypotheticalprotein	5.527807291	0.072133333	2	2	6604117.67	412757.3544
MGG_06530	aspartateaminotransferase	10.5753867	0.1103	3	3	5552294.449	205640.5351
MGG_07718	ATP-dependentRNAhelicaseDRS1	22.22796333	0.0897	6	7	32138969.29	747417.8905
MGG_00845	hypotheticalprotein	37.63747435	0.132266667	9	12	20209147.68	374243.4756
MGG_08840	nucleolarprotein4	49.31419577	0.221633333	12	17	50315370.65	1143531.151
MGG_04422	18SrRNAbiogenesisproteinRCL1	27.61002737	0.2356	6	9	15741532.52	684414.4572
MGG_15536	hypotheticalprotein	6.63481619	0.0119	2	2	1407584.117	11537.57473
MGG_14748	hypotheticalprotein	11.00746297	0.124566667	3	3	3628387.366	226774.2104

MGG_15576	DNArepairproteinrhp51	5.743165009	0.059466667	1	1	37898264.31	2105459.128
MGG_16445	hypotheticalprotein	4.511832225	0.026233333	1	1	2274788.287	78440.97543
MGG_07012	AGC/RSKproteinkinase	7.109477832	0.059733333	2	2	2465211.461	94815.82544
MGG_08908	mRNAturnoverprotein4	7.342814499	0.0903	2	2	1581627.828	143784.348
MGG_04977	60SribosomalproteinL39	4.138154991	0.1961	1	3	38927383.48	19463691.74
MGG_04204	guaninenucleotide-bindingproteinalpha-2subunit	7.296615909	0.050566667	2	2	743216.7669	41289.82039
MGG_05817	nonsense-mediatedmRNAdecayprotein3	36.93512012	0.2222	9	10	20352985.85	782807.1479
MGG_11561	hypotheticalprotein	5.072348799	0.0253	1	1	0	0
MGG_11170	hypotheticalprotein	4.359287639	0.0424	1	1	784274.4012	34098.88701
MGG_03984	54SribosomalproteinL7	14.16448548	0.184766667	4	6	15870411.19	661267.1331
MGG_04085	hypotheticalprotein	30.18594117	0.103766667	7	9	12092735.95	262885.564
MGG_11165	hypotheticalprotein	8.56141225	0.023466667	2	2	82347777.9	1349963.572
MGG_03378	nuclearcondensincomplexsubunitSmc4	11.39332412	0.0254	3	3	24717396.81	274637.7424
MGG_06077	translationinitiationfactoreif-2bsubunitalpha	14.32129094	0.139633333	3	3	1906582.269	100346.4352

MGG_05756	hypotheticalprotein	13.54074073	0.223533333	3	5	7084617.457	506044.1041
MGG_03129	U3smallnucleolarRNA-associatedprotein15	54.93549109	0.2997	13	18	59695400.87	1658205.58
MGG_11779	hypotheticalprotein	64.69072834	0.1191	16	18	103926633.3	895919.2527
MGG_01325	highmobilitygroupprotein	27.4893328	0.260633333	6	7	17151196.15	1429266.346
MGG_00382	GPN-loopGTPase3likeprotein	9.935261708	0.103333333	2	3	6449732.383	586339.3075
